# Emergence of activation or repression in transcriptional control under a fixed molecular context

**DOI:** 10.1073/pnas.2413715122

**Published:** 2025-09-22

**Authors:** Rosa Martinez-Corral, Dhana Friedrich, Robert Frömel, Lars Velten, Jeremy Gunawardena, Angela H. DePace

**Affiliations:** ^a^Department of Systems Biology, Harvard Medical School, Boston, MA 02115; ^b^CRG (Barcelona Collaboratorium for Modelling and Predictive Biology), Barcelona 08003, Spain; ^c^Centre for Genomic Regulation (CRG), The Barcelona Institute of Science and Technology, Barcelona 08003, Spain; ^d^Universitat Pompeu Fabra (UPF), Barcelona 08003, Spain; ^e^Department of Organismic and Evolutionary Biology, Harvard University, Cambridge, MA 02138; ^f^HHMI, Chevy Chase, MD 20815

**Keywords:** transcription factor, duality, nonmonotonicity, gene regulation, nonequilibrium regulation

## Abstract

Transcription factors (TFs) can be both activators and repressors of gene transcription. This can manifest as “duality,” where the transcriptional response increases (activation) with TF concentration in one context but decreases (repression) in another context, or as “nonmonotonicity,” where, in the same context, the response increases in part of the concentration range and decreases outside that range. Here we use biophysical models of gene regulation to investigate how duality and nonmonotonicity relate to the interactions between a TF, Polymerase and the regulatory DNA. We distinguish two modes of TF action on Polymerase: “coherent,” with interactions either positive or negative, and “incoherent,” where interactions are a mix of both. For TFs that act incoherently from a single TF–DNA binding site, nonmonotonicity can arise, but only under nonequilibrium models. For single-site models, we show that nonmonotonicity can never happen under the common thermodynamic models of gene regulation, which consider equilibrium conditions and ignore the dissipative nature of the transcription process. Moreover, we show that merely changing the TF–DNA binding affinity, while keeping other features fixed, can tune the response between activation and repression, with responses either evaluated as a function of TF concentration or site number. Using the mammalian Sp1 as a case study and synthetically designed target sequences, we find experimental evidence for nonmonotonicity, and activation or repression tuned by affinity, which we interpret as evidence of incoherent action. Our work highlights the importance of moving from a TF-centric view to a systems view when reasoning about transcriptional control.

## Significance

Many transcription factors (TFs) can activate and repress gene transcription. Traditionally, this switch in function has been attributed to binding different coregulatory proteins. In this work, we explore a different mechanism, where a TF acts incoherently, simultaneously favoring and hindering transcription. In this case, we show that the TF–DNA binding affinity can tune the response to a TF between activation or repression, without requiring changes in coregulators nor other changes in the system’s molecular composition. Our mathematical models also allow us to clarify how the relationship between TF concentration and expression output is impacted by the underlying molecular mechanisms of transcription and whether processes take place at or away from thermodynamic equilibrium, which is an active area of research.

Transcription factors (TFs) are proteins that regulate transcription through binding to specific sequences in regulatory DNA. TFs are commonly considered to be either activators or repressors, sometimes based on genetics (e.g. removing or disabling the TF) and sometimes based on biochemistry (e.g. titrating the concentration of the TF). However, it is well known that some TFs can behave as either activators or repressors, depending on the targets, cell types, or signaling environments; examples include the *Drosophila* TFs Dorsal (1–4) and KrÃ¼ppel (5–8), and many mammalian TFs, including the glucocorticoid receptor (9), FOXO3 (10), Sp3 (11), Myc (12), Yin-Yang 1 (13, 14), and NF-κB (15, 16). TFs that can behave as either activators or repressors are referred to in the literature as “bifunctional” or “dual” and appear to be widespread (17–20).

**Fig. 1.**
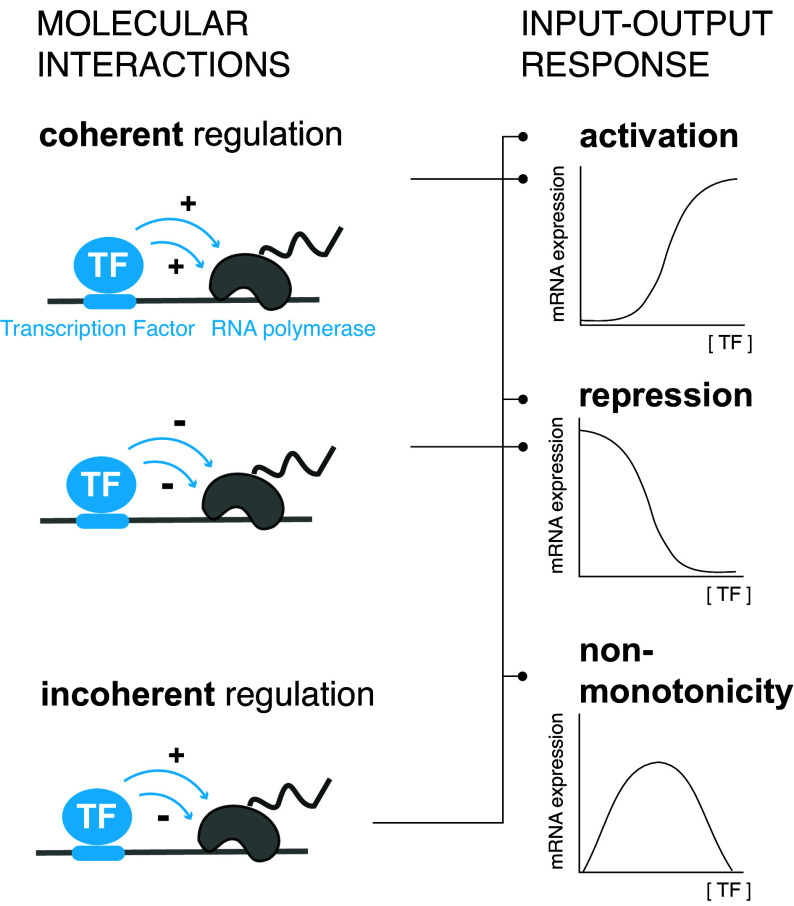
TFs can act coherently or incoherently on RNA Polymerase and this impacts the input–output response. *Left*: We consider two primary types of molecular interactions in this work: Transcription factor (TF)–DNA interactions (in terms of binding site affinity and binding site number), and the interactions between TFs and RNA Polymerase (Pol), which can positively or negatively influence Pol binding or activity. Such TF–Pol interactions are represented in the cartoon with multiple arrows because a TF may modulate various aspects of Pol binding and/or activity. For example, a TF may influence Pol binding, as well as Pol elongation rate. According to such effects, TFs can interact with Pol coherently, where all interactions are either positive or negative, or incoherently, where interactions are both positive and negative. *Right*: Depending on how these interactions are modeled, and whether it is assumed that the molecular processes take place at or away from equilibrium, different types of input–output responses can be observed. The *Top* two schematics (activation and repression) are monotonic, e.g. increasing continuously over a given TF concentration range (activation). The *Bottom* schematic is nonmonotonic, e.g., it increases and then decreases over a given TF concentration range. Coherent regulation always gives rise to the corresponding monotonic responses (horizontal lines), whereas incoherent regulation can give rise to all three types of responses, depending on the configuration of the model (vertical and horizontal lines). TF duality, as defined by the fact that a TF activates or represses over a given concentration range, can be the result of switching between the coherent positive and negative regulatory modes, or reflect incoherent action.

From a biochemical perspective, activation and repression are defined by how the concentration of a TF influences target gene expression: Increasing the concentration of an activator increases transcript levels, whereas increasing the concentration of a repressor reduces them (Fig. 1, *Right* and *Top*-*Middle*). However, TFs can also shift between acting as activators or repressors as their concentration changes, which is called “nonmonotonicity” (e.g. refs. 5 and 21) (Fig. 1, *Bottom*-*Right*). Nonmonotonicity has often been attributed to stress due to TF overexpression (22), or “squelching,” where increasing amounts of TF soak up other regulatory proteins and thus disable activation (23–25). However, nonmonotonicity may arise from the regulatory function of a TF on a target gene (26, 27), and may serve a useful regulatory role in cells, restricting the activation of a target gene over a limited range of input concentrations (27), or providing a means for negative autoregulation (26).

Recent data suggest that nonmonotonicity may also be a signature of underlying mechanisms of gene regulation. Many TF-regulated processes dissipate energy, for example to move nucleosomes around (28). This contrasts with the common assumption that gene regulation can be described by a process that is in equilibrium and does not require energy expenditure (29). An open question in the field is to what extent nonequilibrium regulation must be incorporated into models of transcription in order to adequately capture and predict experimental data, and what might be the regulatory capabilities enabled or enhanced by such energy dissipation (30–33). Recently (27), Mahdavi et al. studied a specific model of gene regulation where a TF binds to a single site and regulates the recruitment of RNA polymerase to the promoter. The study found that this model can produce nonmonotonic responses if the system is away from equilibrium, suggesting that nonmonotonicity may be a signature of nonequilibrium regulation.

Our goal in this paper is to build a conceptual framework to reason about duality and nonmonotonicity at a given target gene, to provide insight into the biophysical mechanisms of gene regulation. Our strategy is to use mathematical modeling to synthesize observations and concepts in the literature into a biologically plausible framework that can make experimentally testable predictions. This strategy requires us to clarify definitions, make multiple types of mathematical models and explore them systematically. Our long-term motivation is to decipher how regulatory evolution in animals is enabled and constrained by the biophysical mechanisms of transcription. From this perspective, it is particularly important to consider the role of TF–DNA binding sites in tuning regulatory responses because TF–DNA binding sites change rapidly over evolutionary time (e.g. refs. 34 and 35). TF–DNA binding site affinity is therefore a primary focus in our analysis of our models.

A common modeling formalism has been to base models on the principles of equilibrium statistical mechanics. In these models, molecules bind on DNA under conditions of thermodynamic equilibrium, in the absence of energy expenditure, and the output transcription of the system is an average of state-specific transcription rates taken over the steady-state probability distribution (29). The key challenge to this modeling formalism is that eukaryotic gene regulation involves multiple processes that dissipate energy, like ATP-dependent chromatin remodeling and posttranslational modifications of the DNA and TFs. Therefore, we will use our models to investigate the impact of nonequilibrium processes on recruiting regulatory molecules to DNA (which could also occur under equilibrium conditions), and to distinguish these effects from the irreversible cycle of producing an mRNA transcript (which is always a nonequilibrium process that dissipates energy) (33, 36).

Our models will consider a TF acting at a single target gene. Based on the vocabulary in the literature of “bifunctional” or “dual” TFs, we define “duality” to be the feature whereby a TF can behave as an activator and a repressor in a given concentration range. TFs have been considered to activate or repress by either favoring or disfavoring the activity of the RNA polymerase (Pol), the machinery that transcribes DNA into RNA, at a given target gene. We call these regulatory modes “coherent positive” or “coherent negative” (Fig. 1, *Left* and *Top*-*Middle*). If regulation is coherent, the switch between activation and repression has commonly been explained in terms of changing molecular context, for example different coregulators recruited by the TF at different target genes (17–19). Alternatively, regulation may be incoherent, with a TF simultaneously acting on Pol in a positive and negative manner (Fig. 1, *Left* and *Bottom*). Thus, a “dual” TF can either be acting coherently and switching between the coherent positive and negative modes, or acting incoherently.

The incoherent regulatory mode was recently proposed to explain data in bacteria (37), and it is plausible that it is more widespread than recognized so far. Many eukaryotic TFs have been described to have activation and repression domains (38–40) or fragments with both activating and repressive activities (20, 22, 41). Despite the common view that TFs only use a subset of their activities at a time, it is plausible that they are in fact simultaneously used, making regulation incoherent. For example, the activity of Bicoid, a classical *Drosophila* activator, increases when a fragment of the protein is deleted (42, 43), which may be interpreted as that part mediating a repressive effect. Moreover, Bicoid was described to interact with both histone acetyltransferases (commonly activating) and histone deacetylases (commonly repressive) (44), and it was recently found to both promote and prevent progression of a gene regulatory system into transcriptionally active states (45).

When regulation is incoherent, whether the response to increased TF concentration is activating or repressive (which we refer to as response “direction”) depends on the strengths of the interactions or the rates of the reactions of the regulatory system. For example, it has been shown that the response direction can be tuned by changes in the strength of the TF–Pol interactions (37), or the strength of the promoter, the docking site for Pol (46). To our knowledge, it has not been examined whether a similar tuning effect can be achieved by the TF–DNA binding affinity, and examining this point is one of the goals of this paper. If the TF–DNA binding affinity can tune the response direction of a TF between activation and repression, this would mean that activation or repression may not depend on any change to how the TF interacts with Pol nor on the molecular partners of the TF, and we expand on the implications of this point in *Discussion*.

The simplicity of models in which a TF acts on a single site can be highly informative (27, 47). However, TFs often bind to multiple sites on a DNA regulatory sequence, especially in animals (48). Therefore, varying site number is relevant for the design of synthetic gene circuits, as well as for the interpretation of natural regulatory sequences within and between species. We examine the relationship between TF binding site number, affinity, and TF concentration within our models. While it is intuitive that all three features should increase the occupancy of the TF on DNA, and influence transcription accordingly, here, we explore the impact of TF incoherence on the response which is more difficult to intuit.

Therefore, in this paper, we investigate the relationships between TF regulatory mode (coherent, incoherent), TF concentration, binding site affinity or number, equilibrium/nonequilibrium assumptions about gene regulation, and the emergence of nonmonotonicity and duality. To this end, we have developed mathematical models and explored how they behave under multiple biologically relevant scenarios. The construction of our models is described in “Model Overview,” and their details and behavior is described in the Results. We analyze the models either analytically or through extensive numerical simulations to identify conditions that prohibit nonmonotonicity, implying that nonmonotonicity may only be possible when those conditions are not satisfied. We also investigate the ability of TF–DNA binding affinity to tune the response between activation and repression, through parameter sets drawn from biologically plausible ranges. We then experimentally investigate the emergence of nonmonotonicity and affinity-dependent activation or repression for the mammalian TF Sp1. In the light of our modeling analyses, the data suggest evidence for incoherent regulation and deviation from equilibrium without any need for fitting models to the data.

## 1. Modeling Overview

We consider various models for how a TF interacts with Pol and therefore regulates transcription. As illustrated in Fig. 1, we encode three kinds of molecular interactions in our models. First, TF–DNA binding and unbinding, thus enabling the study of the effect of varying binding site affinity. Second, the interactions between the TF and Pol. These may either be all positive (coherent positive mode) or all negative (coherent negative mode) (Fig. 1, *Left* and *Top*-*Middle*), or mixed positive and negative (incoherent mode, Fig. 1, *Left* and *Bottom*). Note that in principle TF–Pol interactions can be direct or they can be indirect (49), through coregulators or changes to the chromatin configuration, but that the models do not specify this. Finally, the models also include interactions related to other processes, like the basal assembly of Pol on the promoter (46), or the basal activity of chromatin remodelers.

We consider two broad classes of models. On the one hand, “regulated recruitment models,” which focus on the recruitment of Pol to the DNA by the TFs (32, 50, 51). These models assume that the regulatory sequence of the gene can be in various states depending on what molecules are bound, and the rate of transcription is assumed to be proportional to the steady-state probability of Pol bound on the DNA. These models can be parameterized in two broad ways, in order to correspond to the assumption that they settle into a steady-state of thermodynamic equilibrium (and therefore correspond to the commonly used thermodynamic models of gene regulation) or not. In this way, we can analyze the implications of nonequilibrium regulated recruitment.

It is important to distinguish the effects of nonequilibrium regulated recruitment from the effects that arise due to dissipation in the transcription cycle. To this end, we also consider “transcription cycle models,” which explicitly include dynamic states of Pol that follow each other in a cycle with potentially irreversible transitions, accounting for the effective irreversibility of the process of transcription. The rate of transcription is then assumed to be the flux through this cycle (47, 52). We focus on this class of models when considering the impact of multiple TF–DNA binding sites, because including the cycle adds relevant capabilities to the model and greater generality.

Our models all consider gene regulation as a Markov Process and are analyzed under a common formalism called the “linear framework” (51, 53, 54) (*Materials and Methods* and *SI Appendix*). This approach exploits graph-based methods to express the steady-state probability distribution of the Markov Process in terms of rational functions in the transition rates of the system. For most of our models, this enables proving some results (e.g. the conditions required for monotonicity) independent of parameter values, as well as ruling out particular behaviors for models that share some assumptions (e.g. single site equilibrium models). However, it is not possible to analytically prove results for all of the biological behaviors that we are interested in, e.g. the ability of the TF–DNA affinity to tune the response of a TF. In these cases, we interrogate our models computationally, by exploring a wide range of biologically plausible parameter values (*SI Appendix*, section A) and plotting the behaviors of the models. Such plots can only illustrate how the system behaves qualitatively to demonstrate relevant concepts, because parameter values can be adjusted in multiple ways to modulate the behaviors observed.

Given a model, we can obtain the steady-state transcription rate at a given TF concentration x, $r^{*}\left(x\right)$, and the corresponding response fold-change ($F\left(x\right)=r^{*}\left(x\right)/r^{*}\left(0\right)$). For multisite models, we can also examine the response fold change as a function of site number N. Our central goal is to analyze whether such input–output responses are increasing, decreasing, or nonmonotonic and how this depends on the three kinds of interactions described above, as well as on the assumption of equilibrium or nonequilibrium steady-state.

## 2. Results

**Fig. 2.**
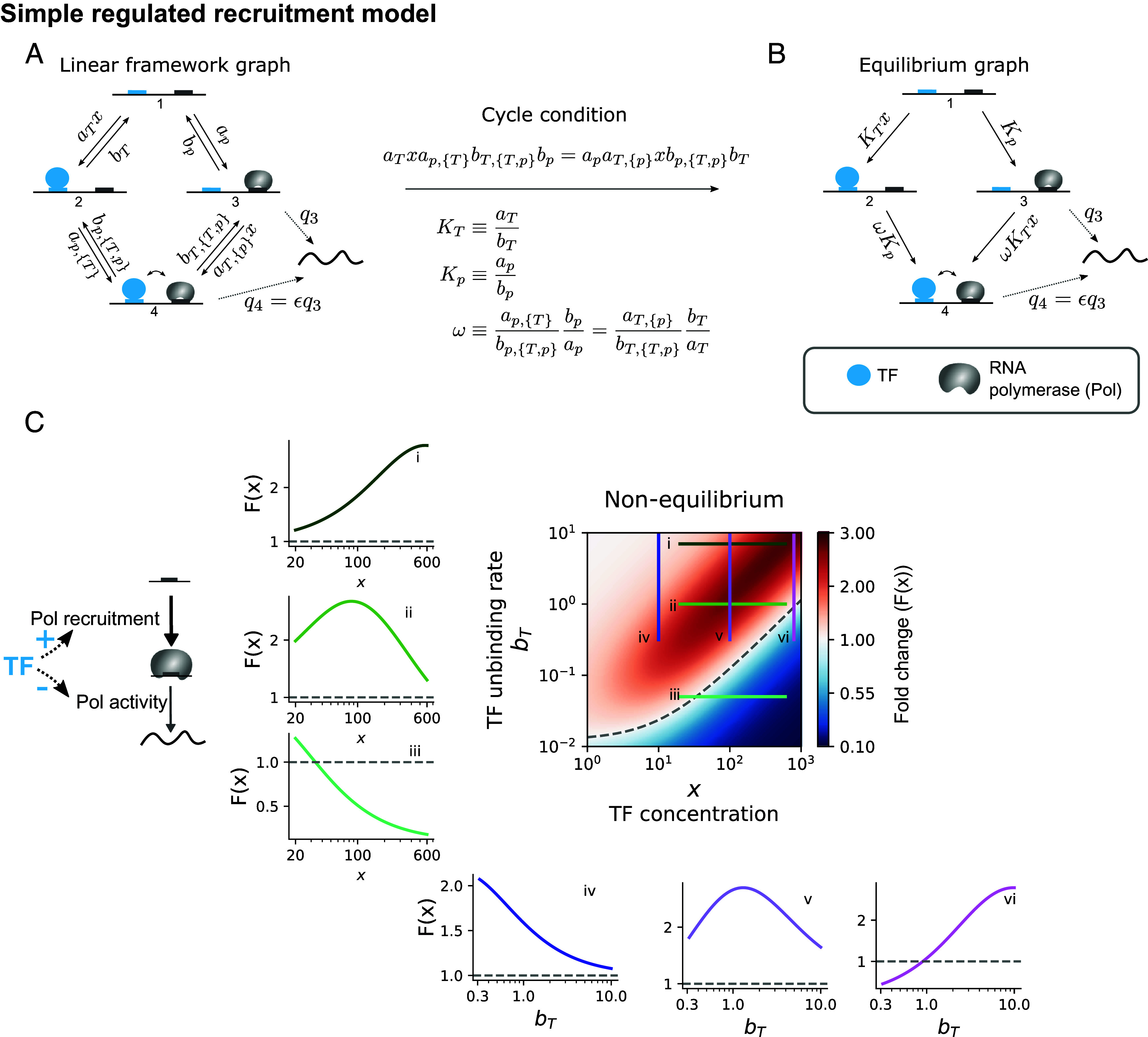
A simple model for TF-regulated Pol recruitment and activity. (*A*) Linear framework graph corresponding to the model, with forward and backward transition rates among states detailed. The TF is assumed to modulate both Pol binding and activity once bound. We use $a_{y,z}$, y∈{T,p}, z∈{{p},{T}} for the binding rate of y to the state with molecules in z bound. If nothing is bound, we omit z. _*T*_ refers to the TF, and _*p*_ to Pol. Similarly, we use $b_{y,z}$ for the unbinding rate of y from the state with molecules in z bound, unless only y is bound, in which case z is omitted. Each state is indexed by an integer, as indicated in the cartoons, and $q_{i}$ is the transcription rate from state i, with ϵ the factor change in $q_{3}$ (basal transcription rate) when the TF is bound. Parameter units: unbinding rates ($b_{T}$, $b_{T,\{T,p\}}$, $b_{p}$, $b_{p,\{T,p\}}$): s^−1^. TF binding rates ($a_{T}$, $a_{T,\{p\}}$): nM^−1^s^−1^. Pol binding rates ($a_{p}$, $a_{p,\{T\}}$): s^−1^ (the concentration term is absorbed into the parameter). (*B*) At thermodynamic equilibrium, the cycle condition holds, and the model in A can be represented just by the corresponding equilibrium graph, with only edges in a single direction detailed (despite transitions still being reversible) with the edge labels given by the ratios between the forward and backward transition rates, as detailed under the arrow in the center of the image. The TF affinity constant $K_{T}$ has units of nM^−1^, $K_{p}$ is dimensionless. ω is also dimensionless and is the binding cooperativity between TF and Pol. (*C*) Colormap of the steady-state response fold change as a function TF unbinding rate ($b_{T}$) and concentration (x), for the model in (*A*), away from thermodynamic equilibrium. The TF acts incoherently: It enhances Pol recruitment but reduces its activity once bound. Lines (*i*–*iii*) illustrate that depending on the TF unbinding rate, the response is either increasing, nonmonotonic, or decreasing over a given concentration range. Lines (*iv*–*vi*) illustrate that a similar behavior occurs at different concentrations, when the response is evaluated as a function of the TF unbinding rate. Parameter values:$a_{T}=0.01$, $a_{T,\{p\}}=0.01$, $b_{T,\{T,p\}}=b_{T}$, $a_{p}=0.01$, $a_{p,\{T\}}=0.1$, $b_{p}=0.1$, $b_{p,\{T,p\}}=0.01$, $q_{3}=1$, and $q_{4}=0.01$.

We begin by considering the well-established recruitment view of transcriptional control (29, 50, 55), where the TF is assumed to modulate the recruitment of the RNA polymerase complex to the gene promoter, as well as its activity once bound (Fig. 2). This model has been commonly studied in the literature (27, 46, 56), and it is helpful to revisit it as a starting point, introduce our modeling approach and build intuition.

The regulatory system can be in four states, distinguished by whether TF and Pol are bound or not. In the light of the linear framework, we define a graph corresponding to the Markov Process (linear framework graph, Fig. 2*A*), with the vertices corresponding to the states of the system and edge labels the transition rates, in dimensions of inverse time. We consider that the steady-state transcription rate $r^{*}\left(x\right)$ is a linear combination of the steady-state probability distribution of the n system states:

[1] $$\begin{array}{c} r^{*}\left(x\right)=\sum_{i=1}^{n}q_{i}P_{i}^{*}\left(x\right), \end{array}$$

where $P_{i}^{*}\left(x\right)$ is the steady-state probability of vertex i at TF concentration x, and $q_{i}$ is the transcription rate from that state. Transcription is assumed to occur only from the states with Pol, at basal rate $q_{3}$, and TF-modulated rate $q_{4}=\epsilon q_{3}$. If ϵ>1, the TF enhances Pol activity, and if ϵ<1, it hinders it.

By assuming linear mRNA decay at rate δ, at steady state the mRNA concentration is given by

[2] $$\begin{array}{c} m^{*}\left(x\right)=\frac{r^{*}\left(x\right)}{\delta } \end{array}$$

and the response fold change by

[3] $$\begin{array}{c} F\left(x\right)=\frac{r^{*}\left(x\right)/\delta }{r^{*}\left(0\right)/\delta }=\frac{r^{*}\left(x\right)}{r^{*}\left(0\right)} \end{array}$$

### 2.1. At Thermodynamic Equilibrium, Responses Are Monotonic for Any Single Site Model.

At thermodynamic equilibrium, the cycle condition must hold (Fig. 2 and *SI Appendix*, section B, Equilibrium steady-states) and the system can be parameterized by just the ratios of forward and backward rates (Fig. 2*B*). This yields the TF–DNA binding affinity $K_{T}$, in units of inverse concentration, the unitless Pol affinity $K_{p}$, which has absorbed the Pol concentration, and the binding cooperativity between the two, ω. ω>1 means that TF favors Pol recruitment, and ω<1 that it disfavors it. Therefore, in this model, the TF regulatory mode is defined by both ω and ϵ (the effect on the transcription rate). The coherent positive mode corresponds to the TF enhancing both Pol recruitment and activity (ω>1 and ϵ>1), the coherent negative to hindering both processes (ω<1 and ϵ<1), and the incoherent mode to one parameter being greater than one and the other less than one.

The steady-state transcription rate, $r^{*}\left(x\right)$, is a rational function in the various parameters and the TF concentration x (*SI Appendix*, section B, Equilibrium steady-states):

[4] $$\begin{array}{c} r^{*}\left(x\right)=\frac{q_{3}\left(K_{p}+\epsilon K_{p}K_{T}\omega x\right)}{1+K_{T}x+K_{p}+K_{p}K_{T}\omega x} \end{array}$$

Whether $r^{*}\left(x\right)$ increases or decreases with TF concentration is determined by whether the derivative of $r^{*}\left(x\right)$ with respect to x is positive or negative. A simple calculation of the derivative of Eq. **4** by the quotient rule shows that the sign of the derivative depends only on the expression

$$\begin{array}{c} K_{p}\omega \left(\epsilon -1\right)+\epsilon \omega -1 \end{array}$$

which is independent of x. Therefore, in the coherent positive mode of regulation (ϵ>1, ω>1) responses are increasing (activating), whereas in the coherent negative mode, responses are decreasing (repressive), as introduced in Fig. 1.

The incoherent mode corresponds to ϵ>1 and ω<1, or the reverse, as described in ref. 46. In this case, the derivative sign depends on the actual values of all parameters appearing in the expression. Thus, as described in ref. 46, a change in the strength of the TF effects (value of ω and/or ϵ), or a change in the basal polymerase affinity $K_{p}$, can tune the overall response between activation and repression, despite the TF acting in incoherent mode always. In this model, the TF–DNA binding affinity $K_{T}$ does not play a role in determining the direction of the response. Nor does the sign of the derivative depend on the TF concentration x, indicating that the response is always monotonic for this model, as previously described (27, 56).

We exploited the generalization capabilities of the linear framework to prove that this monotonicity is not limited to the 4-state single-site model in Fig. 2. In *SI Appendix*, section C, we show that for all models where regulation occurs from a single site, the system is at thermodynamic equilibrium, and the response is a linear combination of the steady-state probabilities of the system, we find that responses are monotonic with respect to TF concentration.

### 2.2. Nonmonotonicity Can Arise Away from Equilibrium, and the Response Can Be Tuned by the TF–DNA Binding Affinity.

When the parameters are chosen in such a way that the cycle condition does not hold, the previous 4-state model settles into a nonequilibrium steady state, where all forward and backward rates (Fig. 2*A*) combine to determine the response function (*SI Appendix*, Eq. **S8**). Mahdavi et al. (27) showed that in this case, responses can be nonmonotonic when the TF acts incoherently through increasing the Pol binding on-rate and off-rate, or decreasing both, without affecting the Pol activation rate. We find that the emergence of nonmonotonicity is not specific to the definition of incoherence in ref. 27; it can also occur when incoherent regulation is defined through opposite effects on the modulation of Pol binding and its activity once bound, as in ref. 46 (Fig. 2*C*-*ii*). Regardless of how incoherence is defined, nonmonotonicity cannot arise under coherent regulation (*SI Appendix*, section D).

In addition, we find that over a fixed range of concentrations, the response can appear monotonically increasing, decreasing, or nonmonotonic depending on the TF–DNA affinity. This is illustrated in panels (*i*–*iii*) in Fig. 2*C*, for a set of parameters taken from biologically relevant order of magnitude estimates (*SI Appendix*, section A). The colormap of Fig. 2*C* shows the response fold-change as a function of TF concentration and the TF unbinding rate ($b_{T}$), which is inversely related to the TF–DNA binding affinity under the assumption of a diffusion-limited binding rate. The colormap shows that the peak of the nonmonotonic responses shifts to higher concentrations with reduced TF–DNA affinity (higher unbinding rate), leading to the response over a certain concentration range shifting between activation and repression depending on the affinity (horizontal lines, *i*–*iii*). Similarly, the direction of the response plotted as a function of the unbinding rate, for a fixed unbinding rate range, can also be tuned by the concentration (vertical lines in the colormap, panels *iv*–*vi*).

The same observation—that when the TF acts incoherently, the TF–DNA binding affinity can tune the response between activation and repression—arises for an expanded regulated recruitment model where we consider that the TF can affect the binding of Pol through two separate processes, for example displacing a nucleosome, and through cooperativity at a given conformation (*SI Appendix*, section E and Figs. S1 and S2). Notably, in this expanded case, even at equilibrium when responses are monotonic, the response can switch between activation and repression as a function of a parameter that can be related to the TF–DNA binding affinity, as detailed in *SI Appendix*, section E and Fig. S1*C*).

### 2.3. Nonmonotonicity May Arise Due to the Dissipation Associated with the Transcription Cycle.

**Fig. 3.**
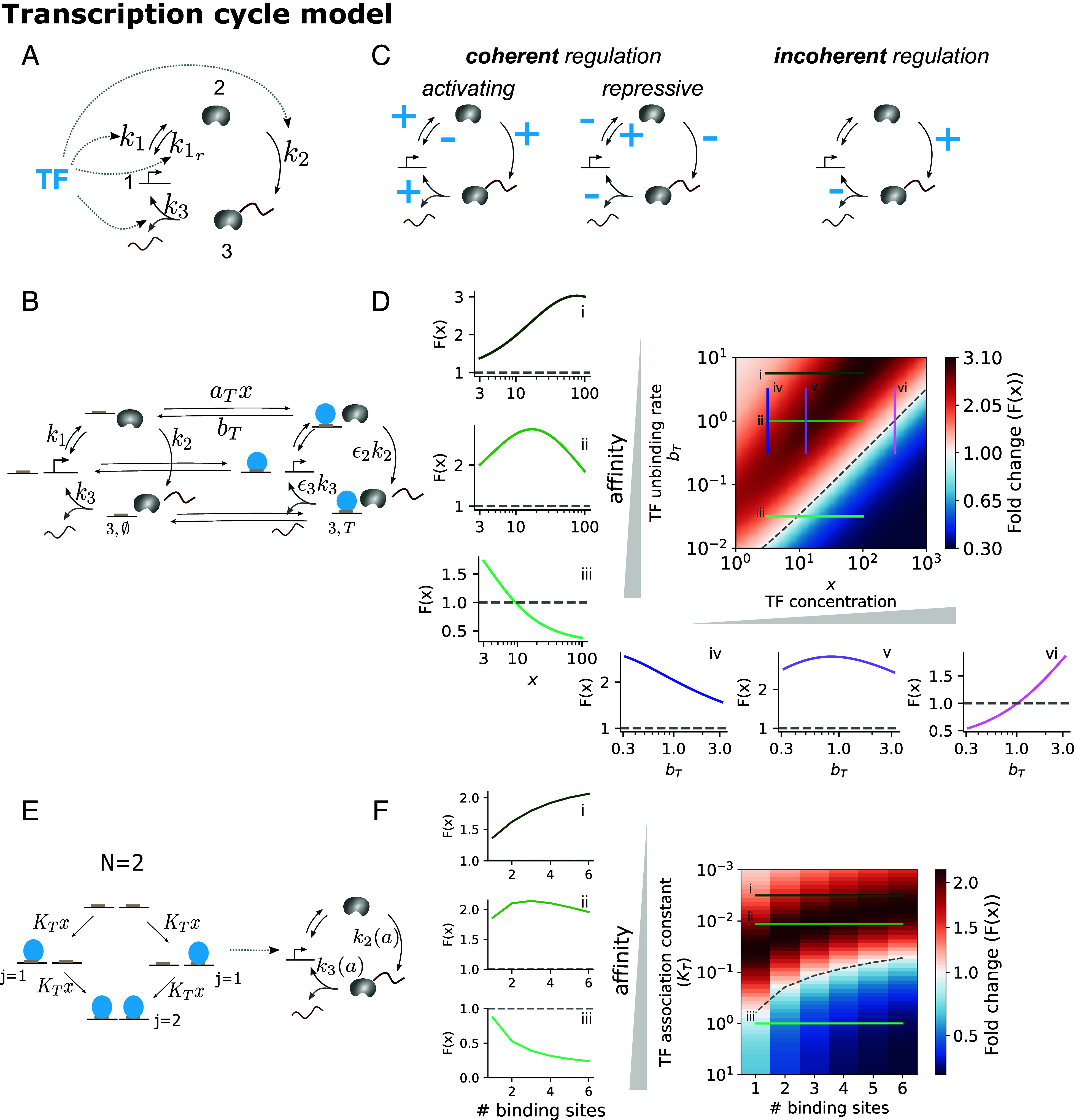
The full range of input–output responses are observed when an incoherent TF regulates the RNA Polymerase transcription cycle. (*A*) Schema of the model: A TF regulates one or multiple transitions of a three-state transcriptional cycle, with each state indexed by an integer i∈{1,2,3}. (*B*) Graph of the model where TF affects a rate while bound. Only specific edge labels are shown for clarity. Each state is now indexed by two elements, an integer that denotes the Pol cycle state, as in (*A*), and a second element that denotes whether TF is bound (i,T) or not (i,∅). Transition rates in the clockwise direction are labeled as $k_{i}$, with i the source state. Transition rates in the counterclockwise direction are labeled as $k_{i,r}$, where r stands for the reverse of rate $k_{i}$. (*C*) Example of regulatory effects corresponding to coherent or incoherent regulation. A plus sign means that the TF accelerates that transition, a minus sign means it decelerates it. (*D*) For the model in (*B*), fold change (F(x)) as a function of the TF unbinding rate and concentration, and corresponding line plots. The TF acts incoherently, accelerating $k_{2}$ ($\epsilon_{2}>1$) and slowing down $k_{3}$ ($\epsilon_{3}<1$). Parameter values: $k_{1}=0.01$ s^−1^, $k_{1,r}=1$ s^−1^, $k_{2}=0.2$ s^−1^, $k_{3}=0.05$ s^−1^, $\epsilon_{2}=15$, $\epsilon_{3}=0.01$, and $a_{T}=0.1$ nM^−1^s^−1^. (*E*) Model where the transcription cycle rates are modulated by the equilibrium average number of bound TF molecules. The graph exemplifies the case of independent binding to N=2 sites. The cycle states and rates are labeled as in (*A*) with an explicit dependence on the equilibrium average number of TF molecules bound (a). (*F*) For the model in (*E*), fold change as a function of the TF affinity constant ($K_{T}$) and number of binding sites, with the lineplots on the *Left* showing fold change as a function of number of sites for three different affinity values $K_{T}$. Parameter values: $k_{1}=0.05$ s^−1^, $k_{1,r}=1$s^−1^, $k_{2,0}=0.2$s^−1^, $s_{2}=1$, $h_{2}=1$, $k_{2,sat}=20$s^−1^, $k_{3,0}=0.05$s^−1^, $s_{3}=0.1$, $h_{3}=1$, and $k_{3,sat}=0.0005$s^−1^. x=2nM. See also *SI Appendix*, Figs. S5 and S6.

It is well known that regulation can also occur downstream of polymerase recruitment, for example at the level of pause-release and elongation (57), and that Pol must leave the promoter for another Pol to bind and initiate another transcript. These processes can be conceptualized in terms of a transcriptional cycle (49), which is effectively irreversible (Fig. 3*A*). It is important to understand to what extent the conclusions from the previous models (affinity-dependent activation and repression, nonmonotonicity when recruitment occurs away from equilibrium) also hold when accounting for nonequilibrium effects downstream of Pol recruitment.

We consider a cycle with three states, as in ref. 47 (Fig. 3*A*). One possible biological interpretation of this simple cycle is unbound polymerase (state 1), bound but inactive polymerase (state 2), and actively transcribing polymerase (state 3), with the latter two transitions irreversible in agreement with experimental observations about polymerase irreversibly escaping the promoter and elongating. The transcription rate is assumed to be proportional to the probability flux from state 3 to state 1 (47):

[5] $$\begin{array}{c} r\left(x\right)=k_{3}P_{3} \end{array}$$

In the first model we consider (Fig. 3*B*), the TF is assumed to bind to and unbind from all three polymerase cycle states with binding rate $a_{T}$ and unbinding rate $b_{T}$. When bound, the TF is assumed to increase or decrease the transitions in the polymerase cycle by a factor $\epsilon_{i}>0$. If the TF enhances (reduces) a given rate, $\epsilon_{i}>1$ (<1) when the TF is bound. Therefore, depending on the $\epsilon_{i}$ parameters, the three modes of TF action or parametric regimes can be defined (Fig. 3*C*).

The transcription rate in this case is given by

[6] $$\begin{array}{c} r\left(x\right)=k_{3}P_{3,\emptyset }+\epsilon_{3}k_{3}P_{3,T} \end{array}$$

As for the regulated recruitment models (*SI Appendix*, sections D and E), we find that the coherent positive mode corresponds to monotonic activation, and the coherent negative mode to monotonic repression (See *SI Appendix*, section F for proof approach). Under incoherent regulation, nonmonotonicity can arise (Fig. 3*D*), also for variations of this model with various points of control of the TF, reversibility patterns, and/or more states (*SI Appendix*, section G and Fig. S4).

Assuming that the first transition of the cycle models Pol recruitment, and keeping $\epsilon_{1}=1$ and $\epsilon_{1r}=1$, we can study whether nonmonotonicity arises even if the cycle formed by TF and Pol binding obeys detailed balance, but the overall system is away from thermodynamic equilibrium due to the irreversibility of the rest of the cycle. As illustrated in Fig. 3*D*, we find parameter sets for which this is indeed the case, and the system behaves very similar to the nonequilibrium regulated recruitment model in Fig. 2*C*, with the response over a given TF concentration range being tunable between activation and repression by the TF unbinding rate. Therefore, nonmonotonicity requires some form of nonequilibrium behavior, but it does not necessarily need to occur at the level of recruitment of molecules on DNA.

### 2.4. Adding More TF–DNA Binding Sites Is Analogous to Increasing TF Concentration When Sites Are Functionally Equivalent.

Until now, we have considered TF binding to a single site, with responses evaluated as a function of TF concentration. As mentioned in Introduction, increasing concentration and site number have conceptually been considered interchangeable when sites are functionally equivalent. Therefore, we aimed to assess whether our findings regarding incoherent regulation and affinity-dependent duality also hold when responses are evaluated as a function of TF binding site number and whether the behavior would qualitatively depend on considering equilibrium binding of the subsystem composed of the TF and Pol or the TF alone. To this end, we considered two extensions of the single-site transcription cycle model, which treat equilibrium binding in two different ways.

First, we extended the model in Fig. 3*B* to have multiple equal sites, so that whenever k sites are bound, the fold-change effect on rate i is $\bar{\epsilon_{i}}=k\epsilon_{i}$. In this case, we can analyze the behavior when the binding of TF and Pol is considered at equilibrium by imposing that the TF regulates $k_{2}$ and $k_{3}$, but not $k_{1}$ nor $k_{1,r}$. We found that even in this case of equilibrium binding combined with the rest of the nonequilibrium transcription cycle, responses could be nonmonotonic with respect to site number under incoherent regulation. Moreover, over a fixed range of site numbers, the response could be tuned between activation and repression by the TF unbinding rate (*SI Appendix*, Fig. S5).

Second, we considered a model that makes the assumption that TF binding is sufficiently fast as to reach thermodynamic equilibrium, and the effects of the TF arise from the equilibrium average occupancy of the TF on DNA (Fig. 3*E*). For a single site with affinity $K_{T}$, the equilibrium average number of TF molecules bound to the DNA, a, is given by $a=\frac{K_{T}x}{1+K_{T}x}$. For N sites, it is given by

[7] $$\begin{array}{c} a=\frac{\Sigma_{i=0}^{N}\left(i\left(\begin{array}{c} N\\ i \end{array}\right){\left(K_{T}x\right)}^{N}\right)}{\Sigma_{i=0}^{N}\left(\left(\begin{array}{c} N\\ i \end{array}\right){\left(K_{T}x\right)}^{N}\right)} \end{array}$$

Note that a increases with either TF concentration (x) or site number N (*SI Appendix*, section I).

We assume that a affects a given rate i from a basal value $k_{i,0}$ to a new value $k_{i}$, following a general, potentially nonlinear and saturable function:

[8] $$\begin{array}{c} k_{i}=k_{i,0}+\left(\epsilon_{i}\right)\left(\frac{a^{h}}{{s_{i}}^{h}+a^{h}}\right) \end{array}$$

with $\epsilon_{i}=k_{i,sat}-k_{i,0}$ and $k_{i,sat}>0$ a theoretical maximum or minimum rate value attainable under that TF.

For this second model, when we considered incoherent regulation ($\epsilon_{2}>1$, $\epsilon_{3}<1$) and a TF acting from a single site (N=1), we found that the map of fold-change in the response as a function of TF–DNA binding affinity and concentration could be very similar to that of Fig. 3*D* (shown in *SI Appendix*, Fig. S6*A*). These results show that equilibrium TF binding coupled to the transcription cycle can also produce nonmonotonic responses. We note that we do not need to assume a large nonlinearity in the effects of the TF on the cycle rates (parameterized by h), although we tended to find more pronounced nonmonotonicity over a shorter TF concentration range with higher h values (*SI Appendix*, Fig. S6*B*, h=2, compare to *SI Appendix*, Fig. S6*A*, with h=1).

Analogously to the effect of TF concentration, we found parameter sets for which responses evaluated as a function of increasing number of sites exhibited an affinity-dependent activation or repression, and nonmonotonicity (Fig. 3*F*). Again, either as a function of increased concentration or site number, responses cannot be nonmonotonic under coherent regulation for this model (*SI Appendix*, section J).

Overall, our modeling results suggest that when a TF regulates more than one process in transcription, irrespective of the exact molecular implementation, we can distinguish between three regimes. The first two regimes, coherent activation and repression, lead to monotonically increasing or decreasing responses, respectively; to switch between the two, the TF must change its mode of action (Fig. 1). The third regime is the incoherent regulatory mode, where the response can be nonmonotonic for nonequilibrium models. This result does not imply that energy dissipation occurs at the level of Pol recruitment, since the irreversibility of the process of transcription itself can lead to nonmonotonicity even when the cycle that includes TF and Pol binding maintains detailed balance. In this regime, the peak of the nonmonotonic response can shift with affinity, so that when evaluated over a fixed input range, the response can be tuned between activation and repression just by changing the TF–DNA binding affinity, either when responses are evaluated as a function of TF concentration or site number.

### 2.5. Experimental Evidence for Nonmonotonicity and Affinity-Dependent Activation or Repression.

As mentioned in the Introduction, experimentally, nonmonotonicity in the effects of a TF has commonly been explained in terms of squelching (23–25), stress (e.g. ref. 22), or difficulties with TF overexpression. Accordingly, during preliminary work for a previous project (47), when we observed nonmonotonic responses, we attributed them to these types of experimental effects. However, the theoretical results presented above suggest an alternative, where nonmonotonicity reflects incoherent regulation by the underlying TF. In the light of our modeling results, we decided to reexamine the behavior of this previous experimental system.

**Fig. 4.**
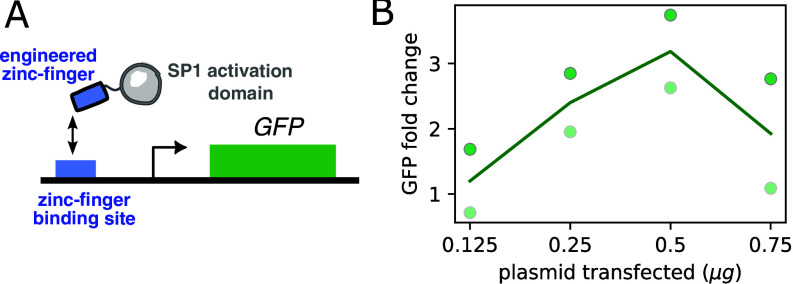
Nonmonotonicity in the response of a reporter regulated by a synthetic ZF-Sp1 TF that binds to a single regulatory site. (*A*) Cartoon of the experimental setup. An engineered zinc-finger was attached to the Sp1 activation domain. A GFP reporter is driven by a single copy of the corresponding zinc-finger binding site. (*B*) The plot shows GFP mRNA fold change, as measured by qPCR, as a function of the amount of transfected TF-encoding plasmids. The line represents the mean of two biological repeats, with the individual datapoints given by the dots, where each shade corresponds to an experiment.

The system is based on a synthetic Sp1 TF fusion acting on a reporter (Fig. 4*A*). More specifically, a domain of Sp1 was fused to an artificially designed zinc-finger DNA binding domain that binds to a (single) 20-bp artificial binding site that does not exist in the mammalian genome, upstream of a CMV promoter that regulates an eGFP reporter. The reporter construct was genomically integrated in a Hek293T cell line (47). Input TF concentration is controlled by transfection, and the transcriptional response by monitoring reporter mRNA levels by qRT-PCR. We have previously shown that concentration of plasmid transfected and TF protein concentration are correlated in this experimental system (*SI Appendix*, Fig. S4*A* in ref. 47). While we do not directly measure TF protein levels in the current experiment, we use TF mRNA levels as a proxy (*SI Appendix*, Fig. S9*A*). We first selected a range of synTF plasmid concentration over which increases in input TF does not affect the expression of GAPDH nor p21 (*SI Appendix*, Fig. S9*B*), which would suggest neither squelching (GAPDH) nor stress (p21) is relevant in this concentration range. Despite this, we could see a nonmonotonic response in the reporter (Fig. 4*B*). In our models of regulation from a single site, we have seen that nonmonotonicity cannot arise in the coherent regimes, nor under a complete equilibrium model (see *SI Appendix* for proofs). In the light of this, and the absence of apparent squelching in our data, we interpret these data as evidence for incoherent regulation that causes nonmonotonicity, and deviation from a purely equilibrium model. However, we cannot pinpoint specific TF-regulated processes, given that our models can exhibit all very similar behavior.

We next analyzed SP1 regulatory activity while keeping its concentration constant at endogenous levels. In this experiment, we varied the number and/or affinity of binding sites -parameters that our models predict can also modulate the direction of the transcriptional response. Because TF concentration remains unaltered, the observed effects are unlikely to be due to squelching. Using the massively parallel reporter assay developed in Frömel et al. (58) [lentiMPRA, (59)], we assayed a library of 276 synthetic regulatory sequences combining 1-6 Sp1 binding sites and 6 affinity ranges (such that all sites in a design corresponded to the same affinity range) in K562 cells. We included different orientations and spacings of sites and used random background DNA. This approach enabled us to characterize the impact of binding site number and affinity on reporter expression, while averaging over the effects of DNA sequence context and spacing and orientation of sites.

**Fig. 5.**
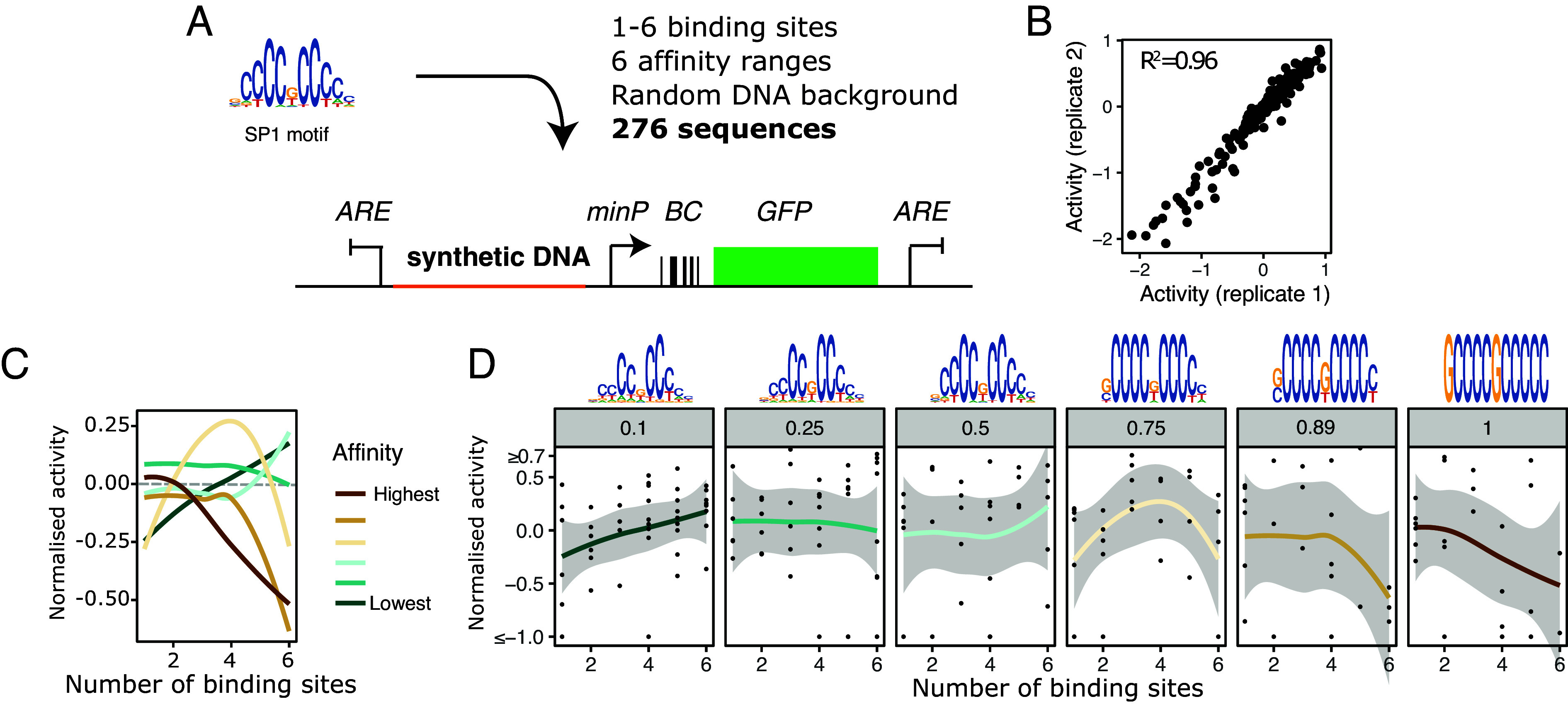
Nonmonotonicity and affinity-dependent activation or repression by Sp1 in K562 cells by lentiMPRA. (*A*) Cartoon of the experimental setup. A library of synthetic regulatory sequences with varying number of SP1 sites, and of different affinities, are placed upstream of a reporter. The constructs are lentivirally integrated into the genome, and responses quantified by RNA/DNA barcode sequence quantification. The protocol follows (59). minP: minimal promoter. BC: barcode. ARE: antirepressor element. (*B*) Correlation of raw activity measures among two technical replicates. (*C* and *D*) Normalized activity as a function of site number, by affinity (0.1: lowest, 1: highest). All sites in a sequence correspond to the same affinity group. Motifs were created by randomly sampling from the PWM such that several instances with a similar likelihood according to the PWM (i.e., a similar affinity) were created. The corresponding likelihood percentiles are given in the subplot titles of panel (*D*). See *Materials and Methods* and ref. 58. (*C*) Smoothed mean activity, averaging over the data corresponding to different orientations, spacings, and random DNA around the sites. (*D*) Smoothed mean activity alongside individual datapoints (corresponding to different orientations, spacings, and random DNA). In (*C* and *D*), the average data lines were plotted with the geomsmooth function of ggplot, with the error representing the 95% CI of the mean. See *SI Appendix*, Fig. S9*D* for nonsmoothed means and medians.

Binding sites were placed upstream of a minimal promoter driving a barcode and GFP expression (Fig. 5*A* and *Materials and Methods*). Activity was quantified as the logarithm of the ratio of RNA-level barcode codes over DNA-level barcode codes for each candidate regulatory element. Comparison of two technical replicates gave a high correlation (Fig. 5*B*), demonstrating a good quality of the data. We further normalized the activity measure by subtracting the mean activity of regulatory elements with only random sequences, so that values above or below 0 correspond to more or less expression as compared to this average activity of random DNA.

When we plotted normalized activity as a function of number of binding sites and stratified by motif affinity, we saw that the average of the data exhibited an affinity-dependent switch, with an activating trend for lower affinities, nonmonotonicity for intermediate affinities and repression for higher affinities (Fig. 5 *C* and *D*, solid lines, *SI Appendix*, Fig. S9*D*), in line with the modeling results in Fig. 3*F*. We note that the dynamic ranges of the responses in the data are small, as we see in our models. Moreover, there is a lot of variability in the data. This is most likely due to the differences in the background sequences in which the Sp1 sites are placed. To test the statistical significance of the affinity-dependent activation or repression, we fitted the data with generalized additive models under shape constraints (60), so that the data in each affinity condition were fitted with a model constrained to being either an increasing, bell-shaped or decreasing function of the number of binding sites. We used the Akaike Information Criterion (AIC), which allows us to compare model fits while penalizing for model complexity, in order to assess whether the fits to any of these functions was better than the fit to a flat line. As shown in *SI Appendix*, Fig. S9*C*, this analysis favored an increasing response at the lowest affinity, a nonmonotonic response at the intermediate affinity group of 0.75, and a decreasing response for the strongest affinity, therefore offering statistical support for an affinity-dependent switch. In the light of our model in Fig. 3*E*, this cannot be explained under coherent regulation (*SI Appendix*, section J), therefore suggesting that incoherent regulation is at play.

## 3. Discussion

For decades, the effects of TFs have been studied through genetic manipulation, demonstrating that many TFs can act as both activators and repressors. This “duality” has commonly been attributed to TFs switching between coherently activating or repressing Pol in a context-dependent manner. However, increasing evidence for TFs acting incoherently, e.g. able to activate and repress simultaneously, requires us to revisit this interpretation. Here, we have used mathematical models to conceptualize how overall activation or repression at the level of the transcriptional response to a TF relate to the interactions between the TF and its target site, the TF, and the transcriptional machinery and basal processes not affected by the TF. Because TFs can act on transcription in multiple ways, our models are built at a coarse level. Along the text we have given some specific molecular interpretations to build biological intuition, but wish to note that other molecular interpretations are possible, for example in the case of the states of the Pol cycle. Regarding how incoherence arises, it could be due to intrinsic biochemical properties of the TF molecule or through interactions with other molecular partners. One such possibility is illustrated in *SI Appendix*, section H–Incoherent regulation through functional interference among activators (*SI Appendix*, Fig. S7). Regardless of the exact molecular implementation, we have conceptualized TF action as either coherent - where the TF only promotes or hinders transcription - or incoherent - where both effects occur simultaneously. We have shown that coherent action from a single site produces monotonic responses, and switching between activation and repression requires a mechanistic change, in line with the classical literature on duality (14, 17–19). In contrast, incoherent regulation allows dual responses without a change in mechanism, depending instead on the balance of various processes. We distinguish three potential ways in which this balance can be tuned while incoherence is maintained. First, by modulating the strength of the positive and negative TF effects, as reported in the work of ref. 37 due to changes to the position of a TF binding site. Second, by changes to factors other than the TF, for example, the concentration of a chromatin regulator (*SI Appendix*, Figs. S1*D* and S2*C*) or promoter strength (46). Finally, by changing the occupancy of TF on DNA, as determined by the binding site affinity, or number of binding sites, which to our knowledge has not been studied before. Previous work has examined the case where multiple TF–DNA binding sites influence the expression of a gene, with distinct sites having different roles (56). In this case, nonmonotonic responses with respect to TF concentration can arise even for systems at thermodynamic equilibrium (56). Yet, we have shown that nonmonotonicity can also arise in a simpler case where there are multiple equivalent TF–DNA binding sites. In this case, we have shown that the response as a function of site number can also be tuned between activation and repression by the TF–DNA binding affinity, similar to responses examined as a function of concentration for single site models. Overall, our findings imply that the response to a TF can change between activation and repression even if the molecular context in which the TF acts (other than its DNA binding sequence) is both qualitatively and quantitatively stable.

Therefore, when designing synthetic sequences, if the TF acts incoherently, binding site number and affinity may be carefully tuned to achieve the desired effect. On the other hand, in natural scenarios duality could be achieved by simple changes to TF–DNA binding sites, rather than through changes in coregulators. Because TF–DNA binding sites are small and degenerate, they can evolve quickly with few mutations (61, 62). Our results raise the possibility that incoherent TFs facilitate rapid regulatory evolution by permitting duality to emerge with relatively few mutations concentrated in regulatory DNA rather than in regulatory proteins themselves, which can circumvent detrimental effects of TF mutations due to pleiotropy (63). We hope these considerations help better experimental design and regulatory sequence interpretation.

We have also examined the link between nonmonotonicity and nonequilibrium regulated recruitment. In previous work by Gedeon et al., it was shown that nonmonotonicity could arise in equilibrium regulated recruitment models with more than one site, where the effects of the TF are not coherent across sites or with higher occupancy of the sites (56). More recently, it has been shown that even with a single site, nonmonotonic responses can arise in a regulated recruitment model away from equilibrium (27) (the model is the same as that in Fig. 2*A*). So, this could be taken as a suggestion that nonmonotonicity may be a signature of underlying nonequilibrium regulated recruitment, a topic of active research (30–33). However, we have seen that this does not need to be the case. Equilibrium TF–Pol–DNA binding coupled to the regulation of the dissipative transcriptional cycle can already account for nonmonotonic responses. So, these results suggest that nonmonotonicity may be better regarded as an indication of incoherent regulation, although formally proving this for any general single site model or multisite model with equal sites remains open for future analyses.

Although our models can be fitted to experimental data as we show in a related manuscript (58), our purpose with Figs. 4 and 5 is to show evidence for incoherence, which does not require any fitting. With regard to the single site data in Fig. 4, *SI Appendix*, section C shows that no matter how complicated, no single-site equilibrium model could be found that fits the data, so equilibrium models would lead to bad fits. Similarly, a nonequilibrium model in coherent mode could neither fit the data according to our analytical or numerical results. In contrast, we observed similar behavior to the data in Fig. 4 for the incoherent models, suggesting that either those or variations could be found that would fit the data well. In our modeling plots, the responses exhibit dynamic ranges around twofold, sometimes less for the nonmonotonic plots, which is smaller than shown in the data in Fig. 4. However, our models can generate nonmonotonic responses with higher dynamic ranges. This is systematically explored for the model in Fig. 3*B* in *SI Appendix*, Fig. S8 (see *SI Appendix*, section K for details). We note, however, that dynamic ranges are always in the few-fold range, in line with our data and the fact that animal TFs exhibit weak effects when acting from a single site (e.g. ref. 47 and 64). Regarding the data in Fig. 5, we note that our multisite model in coherent mode would not fit the data but a model incorporating incoherent regulation could. In line with our interpretation of incoherence, repressive effects for Sp1 have been reported, even at the same gene locus, despite it being considered primarily an activator (65–67). We also interpret the “antagonism” when chaining “dual” TF domains recently reported by Mukund et al. (22) as a result of incoherence, analogous to the repression we see in our incoherent models when increasing binding site number. Similarly, our work is consistent with the CRX regulation reported in ref. 68, where activation or repression depend on a combination of binding site number, affinity, and promoter strength.

In any case, both for our experimental data as well as those of these other works, multiple molecular mechanisms are possible, and disentangling them will require careful and targeted studies. We hope our analyses will motivate this direction of future work. It is tempting to speculate that the incoherent regulation studied here is a broader feature of TFs in natural systems. Indeed, we have identified more instances of duality and affinity-dependent activation or repression as a function of number of sites in an extended MPRA assay in the hematopoietic system (58). If it is indeed the case that in various systems binding site number and affinity can tune the response direction of a TF, this might be another reason behind the widespread presence of low-affinity binding sites in eukaryotic genomes, a well-known fact that is often puzzling to interpret and has often been reasoned in terms of TF specificity (69–72). According to our findings, intermediate or low affinities might enable TFs to behave in the “right” direction, as required to functionally regulate their targets. This would also have evolutionary implications, as it would be easier to evolve the effect of a TF on a target gene by tuning binding site numbers or affinities, rather than evolving new networks of interacting coregulators. At the same time, tuning transcriptional responses from activation to repression via changes in binding site number and affinity also poses questions regarding the robustness of the responses to mutations. We hope that future work on incoherent TF regulation will clarify the molecular underpinnings of this phenomenon as well as its implications for our understanding of genomes and gene regulation.

## 4. Materials and Methods

### 4.1. Model Simulations.

The calculations of the fold change for a given parameter set and TF concentration x involve first calculating the steady state of the model for that parameter set and a TF concentration of 0, then the steady state at x, and then dividing the latter by the former. For equilibrium systems (Fig. 2*B* and *SI Appendix*, Fig. S1), the calculation of the steady-state for a given parameter set and TF concentration is done as follows (30, 73). Choose a reference state (state 1) and assign $\mu_{1}=1$. Then for each state i, calculate $\mu_{i}$ as the product of edge labels (labels of equilibrium graphs, corresponding to edge label ratios of the original linear framework graph) from the reference state to i. The steady state probability of vertex i is then calculated as

[9] $$\begin{array}{c} P_{i}^{*}=\frac{\mu_{i}}{\Sigma_{i}\mu_{i}} \end{array}$$

These calculations are done in Python.

For the nonequilibrium systems corresponding to the models in Figs. 2 and 3, the steady state probability of each vertex i is calculated as

[10] $$\begin{array}{c} P_{i}^{*}=\frac{\rho_{i}}{\Sigma_{i}\rho_{i}}, \end{array}$$

where

[11] $$\begin{array}{c} \rho_{i}=\Sigma_{T\in \Theta_{i}}\Pi_{T}, \end{array}$$

where $\Theta_{i}$ is the set of spanning trees rooted at i, and $\Pi_{T}$ is the product of the edge labels of tree T. The calculations are done in C++, using 100-digit precision floating-point types provided by the GNU MPFR Library through the Boost interface (www.boost.org). Further justification of the procedure and details for the models in *SI Appendix* are given there.

The calculations of the system in Fig. 3*E* were done in Python. The following formula for the steady state mRNA was used (which can be obtained from the procedure just outlined):

[12] $$\begin{array}{c} m=\frac{k_{3}k_{1}k_{2}}{k_{2}k_{3}+k_{3}k_{1r}+k_{1}k_{3}+k_{1}k_{2}} \end{array}$$

From this formula, the basal mRNA level was obtained with the basal rates, and the mRNA level at a given TF affinity, concentration, and site number N was obtained by calculating a (average number of TF molecules bound, Eq. **7**), and subsequently the corresponding rate values, as detailed in the main text (Eq. **8**). In order to calculate a, we calculated the equilibrium steady-state probability of each binding configuration ν, $P_{\nu }^{*}$, following Eq. **9**. We then obtained a as $a=\sum_{i=0}^{N}\left(i\sum_{\#\nu =i}P_{\nu }^{*}\right)$, where #ν means the number of sites bound in state ν, so the internal sum runs over all states ν that have i sites bound.

### 4.2. SynTF Experiment.

#### 4.2.1. Cell culture and transfection.

HEK293FT cells (Thermo Fisher Scientific) with stably integrated eGFP reporter were cultured as described in ref. 47. Transfection of synTF plasmid constructs was performed using Lipofectamine 3000 (Thermo Fisher Scientific). 500,000 cells were plated in 6 cm culture plates and transfected the following day with the corresponding ng of synTF plus single-stranded filler DNA (Thermo Fisher Scientific) to achieve equal amounts of transfected DNA of 1 μg DNA in total. After 24 h cells were harvested and mRNA extracted using the RNeasy Mini Kit (Qiagen).

#### 4.2.2. qRT-PCR.

500 ng extracted total RNA was reverse transcribed into cDNA for each sample using Protoscript II reverse transcriptase (New England Biolabs) and oligo-dT primers (New England Biolabs). Quantitative real-time PCR was performed in triplicates using iTaqUniversal SYBRGreen reagent (Bio-Rad) on a CFX96 PCR machine (Bio-Rad). Primers were used in a final concentration of 243.2 nM. b-actin expression was used as a reference gene for relative quantification of RNA levels, as $2^{Cq_{\mathrm{actin}}-Cq_{\mathrm{target}}}$. For the GFP, fold change is expressed relative to a control condition where no plasmid is transfected (but there is still some GFP expression due to some basal promoter activity). For the ZF, fold change is expressed relative to the lowest input condition.

### 4.3. MPRA Experiment.

#### 4.3.1. Sequence design.

We generated cis-regulatory sequences (CRS) with different designs of Sp1 binding sites, consisting of 1 to 6 binding sites, 3 orientations (only forward, only backward, tandem forward backward), 3 spacings (4, 10, and 20 base pairs), and 6 different affinities. To design binding sites of various affinities, we drew 100,000 random samples from the position weight matrix (PWM) of SP1_MOUSE.H11MO.1.A from hocomoco.v11 (74) and ranked the resulting sequences by the likelihood according to the original PWM. We selected 6 percentile values (10th, 25th, 50th, 75th, 90th, and 100th quantile) and sampled sequences within ±2.5% of each value, except for the highest affinity, where sequences with the highest match were chosen. A total of 100 sequences for each design was generated computationally. TFBS were placed starting from the 3^′^ end of the sequence, and spaces between the TFBS, as well as spaces between the last TFBS and the 5^′^ end, were filled with random nucleotides, for a total length of 232 bases. 15 base pair adaptors were added 5^′^ and 3^′^. The sequences were then screened for motif occurrences of other key hematopoietic transcription factors (the full list is provided in *SI Appendix*, section O) and sequences with strong binding sites for TFs in the background were excluded. In total 276 SP1 sequences were generated, containing all combinations of 1 to 6 binding sites, 3 orientations, 3 spacings, and 6 different affinities. Additionally, 417 sequences containing only random DNA were synthesized.

#### 4.3.2. lentiMPRA experiment.

Oligos were synthesized at Twist Biosciences, for the experimental procedure the lentiMPRA protocol from ref. 59 was followed. Lentivirus was produced in HEK293FT cells combining library plasmids with our cloned inserts (1.64 pM), psPAX2 (1.3 pM), and pMD2.G (0.72 pM) (Addgene: 137725, 12260, and 12259). 6 h transfection was performed with Lipofectamine 3000 following manufactures protocol. Viruses were collected 72 h after transfection and precipitated with sucrose cushion ultracentrifugation (Boroujeni2018-xo). K562 cells were cultured in RPMI media supplemented with 10% FBS and 1% Penicillin-Streptomycin. 2 million K562 cells for both replicates were infected at a high MOI. Infection was stopped after 20 h and cells were collected 3 d after infection. For nucleic acid isolation and library preparation, we followed ref. 59.

#### 4.3.3. Data processing.

GRE-Barcode association of the library and barcode counting at DNA and RNA level was performed using custom Perl scripts following ref. 59. The correlation of the replicates on RNA level and DNA level is 0.990/0.998. RNA counts per CRS were then normalized by DNA counts and we took the natural logarithm of this ratio as a final (raw) activity measure, with a correlation between replicates of 0.948. 197 sequences passed coverage filters. From the raw activity measure, the mean activity of sequences containing only random background DNA was subtracted, to achieve a scale were 0 is the activity induced by random DNA.

## Supplementary Material

Appendix 01 (PDF)

Dataset S01 (CSV)

## Data Availability

All computational data and processed data have been deposited in https://github.com/theobiolab/TFduality.git (75) and https://doi.org/10.5281/zenodo.16994428 (76).
