# Supplementary material for: Emergence of activation or repression in transcriptional control under a fixed molecular context
Source: Proc Natl Acad Sci U S A. 2025 Sep 22;122(39):e2413715122. doi: 10.1073/pnas.2413715122 (PMC12501156; doi:10.1073/pnas.2413715122)
Supplement: Supplementary file 1 — Appendix 01 (PDF) [file pnas.2413715122.sapp.pdf]

Supporting Information for

Emergence of activation or repression in transcriptional control under a fixed molecular context

Rosa Martinez-Corral, Dhana Friedrich, Robert Frömel, Lars Velten, Jeremy Gunawardena, Angela H. DePace

Rosa Martinez-Corral. E-mail:rosa.martinez@crg.eu.
Angela DePace. E-mail: angela.depace@hms.harvard.edu

This PDF file includes:

Supporting text
Figs. S1 to S9
Table S1
Legend for Dataset S1
SI References

Other supporting materials for this manuscript include the following:

Dataset S1

Supporting Information Text

Data and code availability

All the code to reproduce the plots of the paper is found in https://github.com/theobiolab/TFduality.git.

Theoretical methods and calculations

A. Biologically-plausible parameter value ranges. Throughout the manuscript, unless indicated otherwise, we have exemplified the behaviours of our models by taking parameter values from biologically-plausible ranges, in the light of the following considerations.

- TF concentration: Typical TF concentrations are in the few or a few hundred nM (1, 2) although there is variability among TFs, so we have allowed concentrations of up to 1000 nM, which would still be plausible (3).
- TF binding rate: Plausible values can range from about 0.01 $\text{nM}^{-1}\text{s}^{-1}$ to 10 $\text{nM}^{-1}\text{s}^{-1}$ (4–6), and we have considered values within this range.
- TF unbinding rate: We consider that TFs can be bound on DNA over a range that spans fractions of seconds to a bit more than one minute (6), so we have considered a range of 0.01-10 s^{-1}.
- Pol assembly rate: We note that in our models we do not detail Pol concentration as an individual parameter, but this is absorbed into the binding rate. Moreover, in eukaryotes Pol assembly involves the formation of a multi-protein complex, with potentially some very slow steps especially in closed chromatin regions. Therefore, we have considered a range of values that covers waiting times on the order of a few hours (a rate of 0.0003 s^{-1} would correspond to one event every h, which could occur under conditions of very low promoter strength or very low concentration of Pol molecules) to a waiting time of fractions of seconds for very fast scenarios (rates on the order of 10 or 100 s^{-1}).
- Pol disassembly rate: in (4), a value of 10^4s^{-1} was estimated for the bacterial setting, but clearly a much lower value must correspond to promoters of higher strength or under cooperativity with TFs that may also act on the regulatory sequence but may not be considered explicitly in the model. Therefore, we have considered that parameter values corresponding to bound residence times on DNA down to the order of several minutes could be plausible.
- Other transitions: We have taken timescale ranges with waiting times on the hour-minute-second range.
- Factor changes in the transition rates as a result of regulatory interactions: we have tried to be conservative and have taken factor changes of up to 100 fold in the rates, expect in the case of Fig. S5 where we considered that this is the effect when the TF acts from a single site, but can be higher when acting from multiple sites. We note that much higher values of up to exp(40) are plausible under energy-expending conditions (4), and so our factor changes are conservative.

B. Calculating steady states in the linear framework. The mathematical modelling of the paper follows the linear framework, which was introduced in (7) and subsequently developed (8) and applied to gene regulation for example in (9, 10). These references, as well as the more recent review (11), can be consulted for further details and proofs, and here we only repeat the minimum required to understand the present work.

The linear framework assumes a timescale separation between slow and fast components of a biochemical system. The fast components, in our case the various states of the regulatory region or bound Pol, change over time. The slow components, in our case the TFs and RNA Pol, are assumed to be in excess in a buffer, and their interaction (binding) with the fast components does not change their free concentration. In this setting, the fast components evolve over time following a finite-state, continuous-time, time-homogeneous Markov process, which has a corresponding linear framework graph. The vertices of the graph are the states of the system, the edges the transitions between them and the edge labels the infinitesimal transition rates, which can contain the slow components and have dimensions of $(\text{time})^{-1}$. The Master Equation for the time evolution of the probability of each state of the system is then given by:

$$\frac{d\vec{P}}{dt} = \mathcal{L}(G)\vec{P}, \quad \text{[S1]}$$

where G is the graph, $\vec{P}$ is a column vector of state probabilities that sum to 1, and $\mathcal{L}(G)$ is the Laplacian matrix of G. The Laplacian matrix of G is a square matrix where (i, j), $i \neq j$ contains the label from j to i, and the diagonal terms contain the negative of the column sum. In the case of gene regulation, assuming ergodicity, P_i can be interpreted at the single cell and allele level as the average fraction of time the system spends in state i, whereas at the population level it can be interpreted as the fraction of cells in state i at a given time.

For strongly connected graphs as in the case of this paper (every vertex can be reached from any other vertex) the system always tends to a unique steady state ($d\vec{P}/dt = 0$) up to a scalar multiple, which corresponds to the kernel of $\mathcal{L}(G)$. To calculate this steady state, we can exploit the graph, using the Matrix Tree Theorem.

A spanning tree of G is a subgraph that does not contain any cycle (tree) and spans all graph nodes (spanning). A spanning tree T is rooted at node i if it contains a path from any other node to node i. Let Π_T the product of the edge labels of a spanning tree T, Θ_i the set of all spanning trees rooted at i, and ν the set of all the vertices of the graph. A representative steady state $\rho(G)$, can be calculated using the Matrix Tree Theorem, where each component of the steady state vector is given by:

$$\rho_i(G) = \Sigma_{T\in\Theta_i}\Pi_T \qquad \text{[S2]}$$

Because probabilities sum to 1, the steady-state probabilities of each state can be obtained by normalising the previous quantities:

$$P_i^*(G) = \frac{\rho_i}{\Sigma_{j\in\nu}\rho_j} \qquad \text{[S3]}$$

Using this formula, we computed the steady states of the non-equilibrium models of Fig. 2, Fig. S2, Fig. S3, and Fig. 3. Although it is in principle possible to also use it for 5-state cycle graph in Fig. S4 and the models in Fig. S5 and Fig. S7, the number of spanning trees becomes very large as the number of states in the cycle increases or with increasing number of sites. In those cases, for practical reasons we numerically solved for the kernel of the Laplacian using singular value decomposition. This was done with custom C++ code, using the SVD routine in Eigen 3.3.7 and 50-digit precision floating-point types provided by the GNU MPFR Library through the Boost interface (www.boost.org).

For equilibrium graphs, the calculations are substantially simpler, as explained next.

Equilibrium steady-states. When the biochemical system represented by a linear framework graph is assumed to be at thermodynamic equilibrium, the principle of detailed balance must be satisfied. This means that each pair of states (i, j) is independently in flux balance: let P_i^* be the steady state probability of i, P_j^* the steady state probability of j, and $k_{i,j}$ the transition rate from i to j, then $P_i^* k_{i,j} = P_j^* k_{j,i}$. This implies, first, that all transitions must be reversible. Second, over any cycle, the product of the edge labels in the clockwise direction equals the product of the edge labels in the counter-clockwise direction (cycle condition, Fig. 2). As a result, the quantities that specify the steady state are the ratios of forward and backward transition rates (which are related to the free energy difference between the two states), rather than the individual rates (10). In other words, if we multiply by a factor ϵ a given rate, and multiply by the same factor ϵ its reverse rate, the steady state will remain unchanged. Therefore, the system can be represented by what we call the equilibrium graph (Fig. 2).

Let $K_{m,n}$ be the ratio (label ratio) between the transition rates $k_{m,n}$ and $k_{n,m}$ between nodes m and n. Let ℓ_i be any path from a reference node to node i, Π_{ℓ_i} the product of label ratios along this path, and ν the set of nodes in the graph. Then the steady state probability of node i is given by:

$$P_i^*(G) = \frac{\Pi_{l_i}}{\Sigma_{j\in\nu}(\Pi_{l_j})} \qquad \text{[S4]}$$

As an example, this formula has been used to calculate the steady state transcription rate given in Eq. 4 (with the numerator containing the sum of the steady-state probabilities of the nodes with Pol bound, each multiplied by its corresponding transcription rate). In addition to that case, we used this formula for the model in Fig. S1 and to calculate a in the model of Fig. 3D.

As we have noted in previous work in the linear framework, this formulation is equivalent to the steady states obtained using the common statistical mechanics framework exploited by the thermodynamic models of gene regulation (12, 13). This can be seen by considering the relationships between the label ratios $K_{m,n}$ and the free energy difference Δ_ϕ between m and n: $K_{m,n} = \exp\left(\frac{\Delta_\phi}{k_B T}\right)$, with k_B the Boltzmann constant, and T the temperature.

C. Monotonicity of the response for any recruitment model where a TF binds to a single site at thermodynamic equilibrium.

Consider any model that assumes a given TF at concentration x (in excess with respect to the number of binding sites so that x is unaffected by the binding of molecules on DNA) regulates a gene by binding to a single site, the regulatory system is at thermodynamic equilibrium, and the steady-state transcription rate is taken to be a linear combination of the system steady-state probability distribution. No matter how complicated the system is, the response is always monotonic with respect to x.

To see this, consider an arbitrary system with some states with the TF bound and some states without the TF bound. A schematic of such a system is provided in Schema 1. Let Ψ the set of all nodes with TF bound, and Ω the set of all nodes without TF bound. As explained in the previous section, to calculate the steady state of each of the nodes i, we choose a path from a reference node to i. Let's consider the node in red as the reference. Then it is easy to see that any path from it to any node $i \in \Psi$ will be a product of label ratios that contains x one time i.e. will have a form of $\theta_i x$, where θ_i is a constant. Similarly, for any node $j \in \Omega$, the product of edge label ratios will be a constant θ_j (without x). Let $\psi \in \Psi$ the set of all the nodes with TF bound that contribute to expression, and $\omega \in \Omega$ the set of all the nodes without TF bound that contribute to expression. The transcription rate is of the form:

$$r(x) = \frac{A + Bx}{C + Dx} \qquad \text{[S5]}$$

with q_i the transcription rate from node i and $A = \sum_{i \in \omega} q_i \theta_i$, $B = \sum_{i \in \psi} q_i \theta_i$, $C = \sum_{i \in \Omega} \theta_i$, $D = \sum_{i \in \Psi} \theta_i$.

The derivative with respect to TF concentration x is then:

$$\frac{dr(x)}{dx} = \frac{B(C + Dx) - (A + Bx)D}{(C + Dx)^2} = \frac{BC + BDx - AD - BDx}{(C + Dx)^2} = \frac{BC - AD}{(C + Dx)^2} \quad \text{[S6]}$$

Therefore, the sign of the derivative does not depend upon the TF concentration x, and therefore the response is only monotonically increasing or decreasing at equilibrium, depending on the sign of $BC - AD$. Whether the TF acts as an activator or repressor depends upon the various parameters of the system.

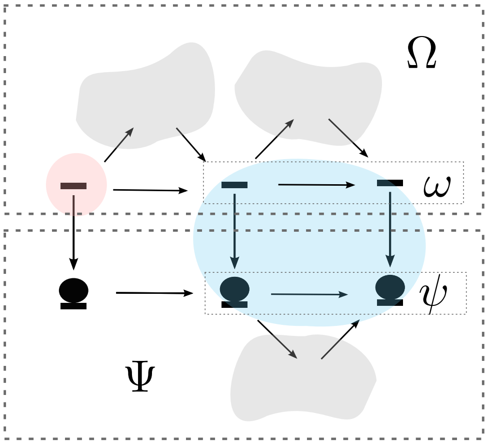

Schema 1. Cartoon that represents an arbitrary gene regulation model where a TF (disc) regulates a gene by binding at a single site, under thermodynamic equilibrium conditions. The states that contribute to expression are inside the blue region. The grey clouds denote arbitrary states and edges between them which do not include binding or unbinding of the TF. All transitions are reversible but edges are only shown in one direction, given that the relevant parameters to determine the steady-state behaviour is the ratio between forward and backward transitions.

D. Monotonicity of the response for the simple regulated recruitment model away from equilibrium under coherent regulation. In the model in Fig. 2A, the TF can modulate the Pol binding rate ($\epsilon_a \equiv a_{p\{T\}}/a_p$ is the TF effect on this process), the corresponding off-rate ($\epsilon_b \equiv b_p/b_{p,\{T,p\}}$ is the effect on this process), and the transcription rate of Pol (the effect is given by ϵ). The steady-state transcription rate $r(x)^*$ is given by:

$$r^*(x) = q_3 P_3^* + \epsilon q_3 P_4^* \quad \text{[S7]}$$

Applying Eq. S3 to calculate P_3^* and P_4^*, $r^*(x)$ can be expressed as:

$$r^*(x) = N/D \quad \text{[S8]}$$

$$N = a_p q_3 \left(a_T b_{T\{T,p\}} \epsilon_a \epsilon_b x + a_p b_{T\{T,p\}} \epsilon_a \epsilon_b + b_T b_p + b_T b_{T\{T,p\}} \epsilon_b + \epsilon \epsilon_b x \left(a_T a_{T\{p\}} \epsilon_a x + a_T b_p \epsilon_a + a_p a_{T\{p\}} \epsilon_a + a_{T\{p\}} b_T \right) \right) \quad \text{[S9]}$$

$$\begin{aligned} D = {} & a_T a_p a_{T\{p\}} \epsilon_a \epsilon_b x^2 + a_T a_p b_p \epsilon_a \epsilon_b x + a_T a_p b_{T\{T,p\}} \epsilon_a \epsilon_b x + a_T a_{T\{p\}} b_p x^2 + a_T b_p^2 x + a_T b_p b_{T\{T,p\}} \epsilon_b x + \\ & + a_p^2 a_{T\{p\}} \epsilon_a \epsilon_b x + a_p^2 b_{T\{T,p\}} \epsilon_a \epsilon_b + a_p a_{T\{p\}} b_T \epsilon_b x + a_p a_{T\{p\}} b_p x + a_p b_T b_p + a_p b_T b_{T\{T,p\}} \epsilon_b + \\ & + a_p b_p b_{T\{T,p\}} \epsilon_a \epsilon_b + a_{T\{p\}} b_T b_p x + b_T b_p^2 + b_T b_p b_{T\{T,p\}} \epsilon_b \end{aligned} \quad \text{[S10]}$$

Differentiating with respect to x gives a fraction (Q/R) whose denominator R is always positive. Therefore, the sign of the derivative is given by the sign of the numerator, which can be expressed as follows:

$$
\begin{aligned}
Q =&(a_T^2 a_p^2 a_{T\{p\}} b_{T\{T,p\}} \epsilon\epsilon_a^2\epsilon_b^2 q_3 - a_T^2 a_p^2 a_{T\{p\}} b_{T\{T,p\}} \epsilon_a^2\epsilon_b^2 q_3+\\
&a_T^2 a_p a_{T\{p\}} b_p b_{T\{T,p\}} \epsilon\epsilon_a\epsilon_b^2 q_3 - a_T^2 a_p a_{T\{p\}} b_p b_{T\{T,p\}}\epsilon_a\epsilon_b q_3+\\
&a_T a_p a_{T\{p\}}^2 b_T b_p \epsilon\epsilon_a\epsilon_b q_3 - a_T a_p a_{T\{p\}}^2 b_T b_p \epsilon\epsilon_b q_3) x^2+\\
&(2a_T a_p^3 a_{T\{p\}} b_{T\{T,p\}} \epsilon\epsilon_a^2\epsilon_b^2 q_3 - 2a_T a_p^3 a_{T\{p\}} b_{T\{T,p\}}\epsilon_a^2\epsilon_b^2 q_3+\\
&2a_T a_p^2 a_{T\{p\}} b_T b_p \epsilon\epsilon_a\epsilon_b q_3 - 2a_T a_p^2 a_{T\{p\}} b_T b_p \epsilon_a\epsilon_b q_3+\\
&2a_T a_p^2 a_{T\{p\}} b_T b_{T\{T,p\}}\epsilon\epsilon_a\epsilon_b^2 q_3 - 2a_T a_p^2 a_{T\{p\}} b_T b_{T\{T,p\}}\epsilon_a\epsilon_b^2 q_3+\\
&2a_T a_p^2 a_{T\{p\}} b_p b_{T\{T,p\}}\epsilon\epsilon_a^2\epsilon_b^2 q_3 - 2a_T a_p^2 a_{T\{p\}} b_p b_{T\{T,p\}}\epsilon_a\epsilon_b q_3+\\
&2a_T a_p a_{T\{p\}} b_T b_p^2 \epsilon\epsilon_a\epsilon_b q_3 - 2a_T a_p a_{T\{p\}} b_T b_p^2 q_3+\\
&2a_T a_p a_{T\{p\}} b_T b_p b_{T\{T,p\}}\epsilon\epsilon_a\epsilon_b^2 q_3 - 2a_T a_p a_{T\{p\}} b_T b_p b_{T\{T,p\}}\epsilon_b q_3)x+\\
&a_T a_p^3 b_p b_{T\{T,p\}}\epsilon\epsilon_a^2\epsilon_b^2 q_3 - a_T a_p^3 b_p b_{T\{T,p\}}\epsilon_a^2\epsilon_b^2 q_3+\\
&a_T a_p^2 b_T b_p^2 \epsilon\epsilon_a\epsilon_b q_3 - a_T a_p^2 b_T b_p^2\epsilon_a\epsilon_b q_3+\\
&a_T a_p^2 b_T b_p b_{T\{T,p\}}\epsilon\epsilon_a\epsilon_b^2 q_3 - a_T a_p^2 b_T b_p b_{T\{T,p\}}\epsilon_a\epsilon_b^2 q_3+\\
&a_T a_p^2 b_p^2 b_{T\{T,p\}}\epsilon\epsilon_a^2\epsilon_b^2 q_3 - a_T a_p^2 b_p^2 b_{T\{T,p\}}\epsilon_a\epsilon_b q_3\\
&a_T a_p^2 b_p b_{T\{T,p\}}^2 \epsilon_a^2\epsilon_b^2 q_3 - a_T a_p^2 b_p b_{T\{T,p\}}^2\epsilon_a\epsilon_b^2 q_3\\
&a_T a_p b_T b_p^3 \epsilon\epsilon_a\epsilon_b q_3 - a_T a_p b_T b_p^3 q_3+\\
&a_T a_p b_T b_p^2 b_{T\{T,p\}}\epsilon\epsilon_a\epsilon_b^2 q_3 + a_T a_p b_T b_p^2 b_{T\{T,p\}}\epsilon_a\epsilon_b q_3 - 2a_T a_p b_T b_p^2 b_{T\{T,p\}}\epsilon_b q_3+\\
&a_T a_p b_T b_p b_{T\{T,p\}}^2\epsilon_a\epsilon_b^2 q_3 - a_T a_p b_T b_p b_{T\{T,p\}}^2\epsilon_b^2 q_3+\\
&a_p^4 a_{T\{p\}} b_{T\{T,p\}}\epsilon\epsilon_a^2\epsilon_b^2 q_3 - a_p^4 a_{T\{p\}} b_{T\{T,p\}}\epsilon_a^2\epsilon_b^2 q_3\\
&a_p^3 a_{T\{p\}} b_T b_p \epsilon\epsilon_a\epsilon_b q_3 - a_p^3 a_{T\{p\}} b_T b_p\epsilon_a\epsilon_b q_3\\
&2a_p^3 a_{T\{p\}} b_T b_{T\{T,p\}}\epsilon\epsilon_a\epsilon_b^2 q_3 - 2a_p^3 a_{T\{p\}} b_T b_{T\{T,p\}}\epsilon_a\epsilon_b^2 q_3\\
&a_p^3 a_{T\{p\}} b_p b_{T\{T,p\}}\epsilon\epsilon_a^2\epsilon_b^2 q_3 - a_p^3 a_{T\{p\}} b_p b_{T\{T,p\}}\epsilon_a\epsilon_b q_3\\
&a_p^2 a_{T\{p\}} b_T^2 b_p \epsilon\epsilon_b q_3 - a_p^2 a_{T\{p\}} b_T^2 b_p \epsilon_b q_3+\\
&a_p^2 a_{T\{p\}} b_T^2 b_{T\{T,p\}}\epsilon\epsilon_b^2 q_3 - a_p^2 a_{T\{p\}} b_T^2 b_{T\{T,p\}}\epsilon_b^2 q_3+\\
&a_p^2 a_{T\{p\}} b_T b_p^2 \epsilon\epsilon_a\epsilon_b q_3 - a_p^2 a_{T\{p\}} b_T b_p^2 q_3+\\
&2a_p^2 a_{T\{p\}} b_T b_p b_{T\{T,p\}}\epsilon\epsilon_a\epsilon_b^2 q_3 - a_p^2 a_{T\{p\}} b_T b_p b_{T\{T,p\}}\epsilon_a\epsilon_b q_3 - a_p^2 a_{T\{p\}} b_T b_p b_{T\{T,p\}}\epsilon_b q_3+\\
&a_p a_{T\{p\}} b_T^2 b_p^2 \epsilon\epsilon_b q_3 - a_p a_{T\{p\}} b_T^2 b_p^2 q_3+\\
&a_p a_{T\{p\}} b_T^2 b_p b_{T\{T,p\}}\epsilon\epsilon_b^2 q_3 - a_p a_{T\{p\}} b_T^2 b_p b_{T\{T,p\}}\epsilon_b q_3
\end{aligned}
$$

It can be seen that pairs or triplets of terms can be made that only differ by factors of ϵ, ϵ_a and/or ϵ_b. The TF acts in coherent positive mode when $\epsilon_a \geq 1$, $\epsilon_b \geq 1$, $\epsilon \geq 1$, with at least two inequalities (if only one, then the concept of coherent or incoherent cannot be applied). In this case, it can be seen that the sign of Q must be positive. Conversely, in the coherent negative mode, the ϵ_i terms are less than one (but positive) and the expression is found to be negative. Therefore, responses are monotonic when the TF acts coherently, activating in the coherent positive mode, and repressive in the coherent negative mode.

E. The TF-chromatin model. Here we expand the model in Fig. 2 so that the TF can modulate Pol recruitment through two molecular processes (Fig. S1A). First, we assume that the transcription start site can exist in two conformations, for which Pol has different affinity, and the TF can modulate the probability of each conformation. The two conformations are interpreted here as the region being either occupied by a nucleosome or free (14, 15), and we will refer to them as closed or open, respectively, although other interpretations are possible. For example, the two conformations could be related to Mediator binding (16). Second, the TF can directly modulate Pol binding through binding cooperativity at a given conformation, similarly to the simple model of Fig. 2. As we did for that model, we want to see how the sign of the interactions between the TF and Pol relate to activation and repression, and how this is modulated by the TF-DNA binding affinity, as well as other parameters in the system. Notably, as a major distinction with the model in Fig. 2, in the equilibrium version of the TF-chromatin model it is not possible to cleanly separate the TF-Pol interactions from the TF-DNA binding interactions, since the opening effect of the TF arises from having a different affinity between the closed and open conformations (17). Away from equilibrium, it is possible to cleanly uncouple the two. We would like to examine the conditions for the behaviours of interest found in other models (monotonicity/non-monotonicity, activation or repression depending on TF-DNA affinity).

E.1. Monotonicity of the response for the TF-chromatin model at equilibrium (Fig. S1B). The steady-state transcription rate for the model in Fig. S1B is given by:

$$r^*(x) = q_3\,P_3^*(x) + q_4\,P_4^*(x) + q_7\,P_7^* + q_8\,P_8^* \qquad \text{[S11]}$$

We consider node 1 in the graph of Fig. S1B as the reference node. Applying Eq. S4, this gives the following equation for $r^*(x)$:

$$r^*(x) = \frac{q_3\,K_p + q_7\,K_t\beta\,K_p + q_4\,K_p\,K_T\omega_c\,x + q_8\,K_t\,\alpha\,K_T\,\beta\,K_p\omega_c\gamma\,x}{1 + K_T x + K_p + K_T K_p\omega_c\,x + K_t + K_t\alpha\,K_T x + K_t\beta\,K_p + K_t\alpha\,K_T\beta\,K_p\omega_c\gamma\,x} \qquad \text{[S12]}$$

We see that this equation conforms to Eq. S5 with

$$A = q_3\,K_p + q_7\,K_t\beta\,K_p \qquad \text{[S13]}$$

$$B = q_4\,K_p\,K_T\omega_c + q_8\,K_t\,\alpha\,K_T\,\beta\,K_p\omega_c\gamma \qquad \text{[S14]}$$

$$C = K_p + K_t\beta\,K_p + 1 + K_t \qquad \text{[S15]}$$

$$D = K_p\,K_T\omega_c + K_t\,\alpha\,K_T\,\beta\,K_p\omega_c\gamma + K_T + K_t\,\alpha\,K_T \qquad \text{[S16]}$$

Therefore, responses for this model are always monotonic at equilibrium. Next, we discuss the parametric conditions for activation and repression.

E.2. Direction of the response to a TF with coherent effects on Pol for the TF-chromatin model at equilibrium. We consider that Pol has a higher affinity for the open conformation ($\beta > 1$). If $\alpha > 1$, $\omega_c > 1$ and $\gamma \geq 1$, all the molecular interactions of the TF enhance Pol binding, indirectly through the effect on the closed/open balance through α, and directly via the binding cooperativity ω_c, with $\gamma \geq 1$ ensuring that the cooperativity effect is in line with the opening effect. If the expression rates (q_i) from each of the Pol-bound states differ, we must also consider their relative values to define the TF mode of action. In this case, an effect of the TF that aligns with the activating conditions for α, ω_c and γ defined above, requires that the transcription rate is at least as high from the Pol-bound states in the open conformation as in the closed conformation, and that the binding of TF either doesn't affect transcription rate or enhances it (otherwise the effect would be inconsistent with the rest and would correspond to incoherent regulation). This corresponds to the following ordering for the q_i: $q_3 \leq q_4 \leq q_7 \leq q_8$. In this case, the TF always behaves as an activator, as shown next.

The condition for the TF behaving as an activator is (c.f. Eq. S6):

$$BC - AD > 0 \qquad \text{[S17]}$$

$$K_pK_T[\omega_c(q_4 + q_8K_t\alpha\beta\gamma)K_p(1+\beta K_t) + \omega_c(q_4 + q_8K_t\alpha\beta\gamma)(1+K_t) - \qquad \text{[S18]}$$

$$-(q_3 + q_7K_t\beta)(1+\alpha K_t) - (q_3 + q_7K_t\beta)K_p\omega_c(1 + K_t\alpha\beta\gamma)] > 0$$

$$\omega_c(q_4 + q_8K_t\alpha\beta\gamma)(1+K_t) - (q_3 + q_7K_t\beta)(1+\alpha K_t) + \qquad \text{[S19]}$$

$$+ K_p\omega_c((q_4 + q_8K_t\alpha\beta\gamma)(1+\beta K_t) - (q_3 + q_7K_t\beta)(1 + K_t\alpha\beta\gamma)) > 0$$

$$q[\omega_c(\epsilon_4 + \epsilon_8K_t\alpha\beta\gamma)(1+K_t) - (1 + \epsilon_7K_t\beta)(1+\alpha K_t) + \qquad \text{[S20]}$$

$$+ K_p\omega_c((\epsilon_4 + \epsilon_8K_t\alpha\beta\gamma)(1+\beta K_t) - (1 + \epsilon_7K_t\beta)(1 + K_t\alpha\beta\gamma))] > 0$$

where we have eliminated a factor of K_pK_T because it doesn't have an impact on the sign, reorganized, and made the following substitutions: $q_3 = q$, $q_4 = \epsilon_4 q$, $q_7 = \epsilon_7\,q$, $q_8 = \epsilon_8\,q$. In order to prove that the TF acts as an activator if all its biochemical activities align, i.e. it promotes chromatin openness and Pol binding ($\alpha > 1$, $\omega_c > 1$, $\gamma \geq 1$), and $\epsilon_i \geq 1$, $\epsilon_j \geq \epsilon_i$ if $j > i$, we will show that each of the lines in Eq. S20 is positive (remember that Pol binding is favored in the open conformation: $\beta > 1$). We begin by the first line (we drop q since it does not impact the sign):

$$\omega_c(\epsilon_4 + \epsilon_8K_t\alpha\beta\gamma)(1+K_t) - (1 + \epsilon_7K_t\beta)(1+\alpha K_t) > 0 \qquad \text{[S21]}$$

$$\omega_c\epsilon_4 - 1 + \omega_c\epsilon_4K_t - \alpha K_t + \omega_c\epsilon_8K_t\alpha\beta\gamma + \omega_c\epsilon_8K_t^2\alpha\beta\gamma - \epsilon_7K_t\beta - \epsilon_7K_t^2\alpha\beta > 0 \qquad \text{[S22]}$$

Given that $\omega_c > 1$, $\epsilon_4 \geq 1$, $\omega_c\epsilon_4 - 1 > 0$. For the rest of the terms in Eq. S22, with $\epsilon_8 = \overline{\epsilon_8}\epsilon_7 \geq 1$ and $\overline{\beta} = \epsilon_7\,\beta > 1$, we have

$$\omega_c\epsilon_4K_t - \alpha K_t + \omega_c\epsilon_8K_t\alpha\beta\gamma + \omega_c\epsilon_8K_t^2\alpha\beta\gamma - \epsilon_7K_t\beta - \epsilon_7K_t^2\alpha\beta > 0 \qquad \text{[S23]}$$

$$\omega_c\epsilon_8K_t\alpha\beta\gamma - \epsilon_7K_t\alpha\beta + \omega_c\epsilon_4 + \omega_c\gamma\overline{\epsilon_8}(\overline{\beta}\alpha) - \alpha - \overline{\beta} > 0 \qquad \text{[S24]}$$

$$[\alpha\beta(\omega_c\epsilon_8K_t\gamma - \epsilon_7K_t)] + [\omega_c\epsilon_4 + \omega_c\gamma\overline{\epsilon_8}(\overline{\beta}\alpha) - (\alpha + \overline{\beta})] > 0 \qquad \text{[S25]}$$

It is easy to see that the terms inside the first square brackets result in a positive term. To see that the same happens for the second square bracket, note that $1 + \overline{\beta}\alpha > \alpha + \overline{\beta}$ if $\alpha > 1$ and $\overline{\beta} > 1$. To see this, let $\alpha = 1 + \theta_a$ and $\overline{\beta} = 1 + \theta_b$, with $\theta_a > 0$, $\theta_b > 0$, then:

$$1 + (1+\theta_a)(1+\theta_b) > (1+\theta_a) + (1+\theta_b) \qquad \text{[S26]}$$

$$1 + 1 + \theta_a + \theta_b + \theta_a\theta_b > 1 + \theta_a + 1 + \theta_b \qquad \text{[S27]}$$

$$\theta_a\theta_b > 0 \qquad \text{[S28]}$$

With a similar reasoning we can show that the second line of Eq. S20 is positive under the parametric assumptions considered:

$$\epsilon_4 - 1 + \epsilon_4 \beta K_t + \epsilon_8 K_t \alpha\beta\gamma - K_t \alpha\beta\gamma - \epsilon_7 K_t \beta + \epsilon_8 K_t^2 \beta^2 \alpha\gamma - \epsilon_7 K_t^2 \beta^2 \alpha\gamma > 0 \quad \text{[S29]}$$

Clearly $\epsilon_4 - 1 \geq 1$ and $\epsilon_8 K_t^2 \beta^2 \alpha\gamma \geq \epsilon_7 K_t^2 \beta^2 \alpha\gamma$ since $\epsilon_8 \geq \epsilon_7$. For the rest, dropping one factor of $K_t\beta$ per term:

$$\epsilon_4 + \epsilon_8(\alpha\gamma) - (\epsilon_7 + \alpha\gamma) > 0 \quad \text{[S30]}$$

Since $\epsilon_8 \geq \epsilon_7$ and $\epsilon_4 \geq 1$, positivity follows seeing that the expression is at least of the form $1 + ab > a + b$ with $a \equiv \epsilon_8$ and $b \equiv \alpha\gamma$.

Repression is ensured ($BC - AD < 0$) if, as before, Pol has higher affinity for the open conformation ($\beta > 1$), but the TF hinders Pol binding so that $0 < \alpha < 1$, $0 < \omega_c < 1$, $0 < \gamma < 1$, and expression is either equal from all Pol bound states ($\epsilon_4 = 1, \epsilon_7 = 1, \epsilon_8 = 1$), or higher from the open conformation but reduced in the presence of TF ($0 < \epsilon_4 < 1$, $1 < \epsilon_8 < \epsilon_7$).

Let's start with the case $\epsilon_i = 1$. In this case, the second line in Eq. S20 vanishes. To see that the first line is negative, notice that in Eq. S22, $\omega_c \epsilon_4 - 1 < 0$ if $0 < \omega_c < 1$ and $\epsilon_4 = 1$. For the rest, let's focus on the left-hand side of Eq. S25. Notice that $1 + ab < a + b$ if $0 < a < 1$, $b > 1$, which can be easily shown if $a \equiv 1 - \theta_a, 0 < \theta_a < 1$, $b \equiv 1 + \theta_b, \theta_b > 0$:

$$1 + (1 - \theta_a)(1 + \theta_b) < (1 - \theta_a) + (1 + \theta_b) \quad \text{[S31]}$$

$$1 + 1 - \theta_a + \theta_b - \theta_a\theta_b < 1 - \theta_a + 1 + \theta_b \quad \text{[S32]}$$

$$2 - \theta_a + \theta_b - \theta_a\theta_b < 2 - \theta_a + \theta_b \quad \text{[S33]}$$

It is then easy to see that the term in the second square brackets in Eq. S25 must be negative. For the other terms, it is easy to see that they are also negative.

When the ϵ_i are no longer 1 but satisfy the constraints mentioned ($0 < \epsilon_4 < 1$, $1 < \epsilon_8 < \epsilon_7$) (now $0 < \overline{\epsilon_8} < 1$) similar reasonings allow to show that both lines in the left-hand side of Eq. S20 are negative.

Behaviour of the equilibrium TF-chromatin model in incoherent regime. The incoherent mode can be encoded in various ways. For example, it may correspond to the TF enhancing the open conformation but exhibiting negative cooperativity with Pol, a misalignment of cooperativities between conformations, or effects on the transcription rates q_i that do not follow the effects on chromatin openness and TF binding. In any of these cases, the above conditions for monotonic activation or repression will not hold. Instead, relationships between the various parameters determine whether increases in TF concentration result in more or less transcription.

As shown in Fig. S1C, when the TF has a higher affinity for the open than closed conformation, therefore promoting the open conformation, but has negative cooperativity with Pol at each conformation, activation or repression behaviours depend on the affinity of the TF for the open conformation relative to that for the closed conformation (α). If the affinity for the open conformation is only a bit better than that for the closed conformation, such that the opening effect is small, the repressive effects dominate and the TF behaves as a repressor, but it can switch to activating for higher differences in the TF affinity for the open conformation relative to that for the closed.

On the other hand, we see that the basal openness of the chromatin, given by K_t, can also tune the response between activation and repression (Fig. S1D). This is an instance of duality caused by a change in a basal regulatory process, analogously to the duality caused by a change in promoter strength described by (18).

Finally, Fig. S1E illustrates duality due to changes in the values of the transcription rate, when the TF effects at this level misalign with those on the Pol binding. Fig. S1F illustrates the effects of inconsistent cooperativities across conformations.

E.3. The TF-chromatin model away from equilibrium due to dissipation in the opening step. We now consider the TF-chromatin model away from thermodynamic equilibrium. We first consider a simplified non-equilibrium model (Fig. S2) and we will then consider the full non-equilibrium model (Fig. S3).

In the model in Fig. S2A, we consider that the transitions between the closed and open states with TF bound are driven by an energy-dissipative process. This could reflect, for example, opening due to ATP-dependent chromatin remodellers. The factor increase in the opening rate when the TF is bound relative to when it is not bound is given by α. For simplicity, we consider that the affinity of the TF does not change among conformations. This could be reasonable if the nucleosome that the TF remodels occupies the Pol binding site but not that of the TF, or the TF has a similar ability to bind closed and open chromatin, as is the case for pioneer TFs. As before, we allow equilibrium binding cooperativity between the TF and Pol at each conformation, which could be due to direct protein-protein interactions, or through co-regulators. With these considerations, the cycle condition is broken for those cycles that contain an opening transition among TF-bound states, but otherwise the cycle condition holds (cycles encompassing only the binding and unbinding transitions ($\{1, 2, 3, 4\}$ and $\{5, 6, 7, 8\}$, as well as the cycle with only Pol bound and covering the closed and open conformations ($\{1, 3, 5, 7\}$).

As in the equilibrium counterpart, there is a cooperativity parameter ω_c, which is modelled as modifying the unbinding rate of the TF and Pol from the states with both molecules bound (4 and 8). γ accounts for a potential difference in the binding cooperativity between the two conformations. Positive binding cooperativity, which favors binding, is given by $\omega_c > 1$, and negative binding cooperativity is given by $0 < \omega_c < 1$. The overall transcription rate is assumed to be given by the weighted average of the transcription rate from each state with Pol bound.

Monotonicity of the response. Considering the previously-stated constraints on the parameter relationships, here we present the reasoning for analytically proving the conditions for monotonic responses under coherent regulation. For the general case without constraints (Fig. S3) we performed an extensive numerical exploration, as explained in the next subsection. Note that in order to simplify the notation, we will now use P_i instead of P_i^* to refer to the steady-state probability of state i.

Coherent positive mode (activation). It can be proved for the non-equilibrium model in Fig. S2A that if $\alpha > 1$, $0 < \beta < 1$, $\omega_c > 1$, $\gamma \geq 1$, and $q_3 = q_4 = q_7 = q_8 = 1$, then

$$\frac{d(P_3(x) + P_4(x) + P_7(x) + P_8(x))}{dx} > 0 \tag{S34}$$

which means that the response to the TF is monotonically increasing when Pol has higher affinity for the open conformation ($\beta < 1$), the TF enhances Pol binding both by $\alpha > 1$ and $\omega_c > 1$, $\gamma \geq 1$, and expression is the same from all Pol-bound states.

In this case, there are a large number of terms in the corresponding gene regulatory function and derivative. In order to show monotonicity, we use the following observation:

Consider two polynomials in x of degree n, and their corresponding derivatives ($'$ denotes derivative with respect to x):

$$A = \sum_{k=0}^{n} a_k x^k \to A' = \sum_{k=1}^{n} a_k\, k\, x^{k-1} \tag{S35}$$

$$B = \sum_{k=0}^{n} b_k x^k \to B' = \sum_{k=1}^{n} b_k\, k\, x^{k-1} \tag{S36}$$

Now consider the derivative with respect to x of the ratio A/B:

$$\left(\frac{A}{B}\right)' = \frac{1}{B^2}(A'\,B - A\,B') \tag{S37}$$

The sign is determined by the term in the parenthesis:

$$Q = A'\,B - A\,B' = \sum_{k=1}^{n} a_k\, k\, x^{k-1} \sum_{k=0}^{n} b_k x^k - \sum_{k=0}^{n} a_k x^k \sum_{k=1}^{n} b_k\, k\, x^{k-1} \tag{S38}$$

Expanding it and rearranging, Q can be expressed as:

$$\begin{aligned}
Q = 0\,(...) - a_0(b_1 + 2\,b_2 x + 3\,b_3 x^2 + ... + n\,b_n x^{n-1}) + \\
a_1\,(b_0 + b_1\,x + b_2\,x^2 + ... + b_{n-1}x^{n-1} + b_n\,x^n) - a_1\,x(b_1 + 2\,b_2 x + 3\,b_3 x^2 + ... + n\,b_n x^{n-1}) + \\
2\,a_2\,x\,(b_0 + b_1\,x + b_2\,x^2 + ... + b_{n-1}x^{n-1} + b_n\,x^n) - a_2 x^2\,(b_1 + 2\,b_2 x + 3\,b_3 x^2 + ... + n\,b_n x^{n-1}) + \\
3\,a_3\,x^2\,(b_0 + b_1\,x + b_2\,x^2 + ... + b_{n-1}x^{n-1} + b_n\,x^n) - a_3 x^3\,(b_1 + 2\,b_2 x + 3\,b_3 x^2 + ... + n\,b_n x^{n-1}) + \\
\vdots \\
(n-1)\,a_{n-1}\,x^{n-2}\,(b_0 + b_1\,x + b_2\,x^2 + ... + b_{n-1}x^{n-1} + b_n\,x^n) - a_{n-1}x^{n-1}(b_1 + 2\,b_2 x + 3\,b_3 x^2 + ... + n\,b_n x^{n-1}) + \\
n\,a_n\,x^{n-1}\,(b_0 + b_1\,x + b_2\,x^2 + ... + b_{n-1}x^{n-1} + b_n\,x^n) - a_n x^n(b_1 + 2\,b_2 x + 3\,b_3 x^2 + ... + n\,b_n x^{n-1})
\end{aligned}$$

Terms can be collected according to the subindices of the coefficients a and b and the exponent of x. For example:

- Subindices 0 and 1, exponent 0: $b_0\,a_1 - a_0\,b_1$

- 1 and 2, exponent 2: $a_1\,b_2\,x^2 - a_1\,x\,2\,b_2\,x + 2\,a_2\,x\,b_1\,x - a_2\,x^2\,b_1 = (b_1\,a_2 - a_1\,b_2)x^2$
- 2 and n-1, exponent n: $2\,a_2\,x\,b_{n-1}x^{n-1} - a_2\,x^2(n-1)b_{n-1}x^{n-2} + (n-1)a_{n-1}x^{n-2}b_2\,x^2 - 2a_{n-1}x^{n-1}b_2\,x = (n-1-2)(b_2\,a_{n-1} - a_2\,b_{n-1})x^n$

We notice that the terms where the subindices are the same cancel, and so the above grouping leads to the following expression for Q:

$$Q = \sum_{i=0...n-1, j=1...n, i<j} (j-i)(b_i\,a_j - a_i\,b_j)x^{i+j-1} \tag{S39}$$

Thus, the derivative is **positive** if

$$b_i\,a_j - a_i\,b_j > 0 \quad \forall\{i,j\}, (i<j) \tag{S40}$$

In this case, or if some terms vanish and others fulfill the condition, monotonicity is ensured.

We have implemented an algorithm that (for the graph in Fig. S2A):

1) Computes the spanning trees rooted at each node and from those, the polynomials for the numerator and denominator of the steady-state response function $r^*(x)$.

2) For each pair of i, j, $i = 0, ..., 3$, $j = 1, ..., 4$ computes the corresponding $c = b_i\, a_j - a_i\, b_j$.

3) Splits each c into expressions with a common factor in $a_T, b_T, a_p, b_p, k_o, k_c$.

This results into a total of 2673 expressions in $\alpha, \beta, \gamma, \omega_c$. We use mathematica to check that each of them is individually positive for the parameter constraints described above, and therefore the whole derivative is positive. Note that in the code for the proofs, ω and γ represent $1/\omega_c$ and $1/\gamma$ in the paper. Therefore the mathematica code requires $\omega_c < 1$, $\gamma \leq 1$ to represent activating conditions.

Using the same approach, we can show that

$$\frac{d(P_8(x))}{dx} > 0 \quad [S41]$$

$$\frac{d(P_7(x) + P_8(x))}{dx} > 0 \quad [S42]$$

$$\frac{d(P_4(x) + P_7(x) + P_8(x))}{dx} > 0 \quad [S43]$$

for the same parametric constraints for α, β, ω_c, γ.

Notably, Eq. S34 and Eqs. S41- S43 imply that the response is also monotonic for the more general case where expression is not the same from all states, but increases with the TF bound and the open conformation such that $q_3 \leq q_4 \leq q_7 \leq q_8$.

To see this, first note that showing that

$$\frac{d(q_3 P_3(x) + q_4 P_4(x) + q_7 P_7(x) + q_8 P_8(x))}{dx} > 0 \quad [S44]$$

if $q_3 \leq q_4 \leq q_7 \leq q_8$ is equivalent to showing that

$$\frac{d(q_3 P_3(x) + q_3\epsilon_1 P_4(x) + q_3\epsilon_1\epsilon_2 P_7(x) + q_3\epsilon_1\epsilon_2\epsilon_3 P_8(x))}{dx} > 0 \quad [S45]$$

for $\epsilon_i \geq 1 \forall i$, $q_3 > 0$. In turn, this is equivalent to showing that

$$\frac{d(P_3(x) + \epsilon_1 P_4(x) + \epsilon_1\epsilon_2 P_7(x) + \epsilon_1\epsilon_2\epsilon_3 P_8(x))}{dx} > 0 \quad [S46]$$

for $\epsilon_i \geq 1 \forall i$.

Let $P_i' \equiv dP_i(x)/dx$. From Eq. S34:

$$P_3' + P_4' + P_7' + P_8' > 0 \quad [S47]$$

$$P_4' + P_7' + P_8' > -P_3' \quad [S48]$$

$$\epsilon_1(P_4' + P_7' + P_8') > -P_3' \quad [S49]$$

Where the last inequality must hold given $\epsilon_1 \geq 1$ and Eq. S43. Moreover, given Eq. S42

$$\epsilon_1(P_7' + P_8') > -\epsilon_1 P_4' - P_3' \quad [S50]$$

$$\epsilon_2(\epsilon_1(P_7' + P_8')) > -\epsilon_1 P_4' - P_3' \quad [S51]$$

$$P_3' + \epsilon_1 P_4' + \epsilon_1\epsilon_2 P_7' + \epsilon_1\epsilon_2 P_8' > 0 \quad [S52]$$

Finally, since we also know $P_8' > 0$, Eq. S46 must hold for $\epsilon_3 \geq 1$.

Coherent negative mode (repression). The same reasoning and algorithm can be used to proof the monotonically decreasing conditions.

A repressive TF can be encoded in the model as reducing the opening rate ($\alpha < 1$) and having negative cooperativity with Pol ($\omega_c < 1$), with $\gamma = 1$. Under these conditions, it can be shown that:

$$\frac{d(P_7(x))}{dx} < 0 \quad [S53]$$

$$\frac{d(P_7(x) + P_8(x))}{dx} < 0 \quad [S54]$$

$$\frac{d(P_3(x) + P_7(x) + P_8(x))}{dx} < 0 \quad [S55]$$

$$\frac{d(P_4(x) + P_3(x) + P_7(x) + P_8(x))}{dx} < 0 \quad [S56]$$

If the repressive role of the TF is also manifested in the expression rates, such that $q_4 \leq q_3 \leq q_8 \leq q_7$ the response continues to be monotonically decreasing, that is

$$q_4 P_4' + q_4\epsilon_1 P_3' + q_4\epsilon_1\epsilon_2 P_8' + q_4\epsilon_1\epsilon_2\epsilon_3 P_7' < 0 \quad \text{[S57]}$$

with $\epsilon_i \geq 1$.

To verify this, note that we can do the same reasoning as above given Eqs. S53-S56:

$$P_4' + P_3' + P_8' + P_7' < 0 \quad \text{[S58]}$$
$$P_3' + P_8' + P_7' < -P_4' \quad \text{[S59]}$$
$$\epsilon_1(P_3' + P_8' + P_7') < -P_4' \quad \text{[S60]}$$
$$\epsilon_2(\epsilon_1(P_8' + P_7')) < -P_4' - \epsilon_1 P_3' \quad \text{[S61]}$$
$$\epsilon_1\epsilon_2\epsilon_3 P_7' < -P_4' - \epsilon_1 P_3' - \epsilon_1\epsilon_2 P_8' \quad \text{[S62]}$$

We note that when $q_3 = q_4 = q_7 = q_8$ and $\gamma \neq 1$, or $\gamma = 1$ but the expression rates are not equal nor satisfy the ordering considered in the above discussion (or $\gamma \neq 1$ and the expression rates are not equal nor satisfy the orderings) then activation or repression is not ensured only on the basis of α and ω_c, and non-monotonic responses can appear even if both of these parameters align (Fig. S2D).

Behaviour in incoherent regime. Contrary to the equilibrium version of this model, in this case the TF opening effect can be completely uncoupled from the affinities of the TF for the two conformations. In this case, when the TF acts incoherently, we find parameter sets for which there is an interplay between the TF unbinding rate and the TF concentration very similar to that of the simple regulated recruitment model plotted in Fig. 2C. As seen in Fig. S2B, the response can be non-monotonic, and for a fixed concentration range, the response can switch between activation and repression due to a change in the unbinding rate.

On the other hand, similar to the equilibrium counterpart, tuning the basal chromatin closing rate can also cause a switch in the response (Fig. S2C). We note that in this model, it is possible to find non-monotonic responses where the expression first goes down with concentration and then up, as in the example given in Fig. S2D-bottom. In this case, this parameter set may be more reasonable if the two conformations are considered in terms of a co-regulator bound or not, which could be another interpretation for this model as we mentioned when introducing the model. In any case, we show that there is not a fundamental limit that precludes responses of this U-shape kind.

E.4. The general non-equilibrium TF-chromatin model. In the previous subsection, we discussed the TF-chromatin model under certain constraints imposing the cycle condition in the Pol effects on chromatin, and cooperativity being dictated only by a change in the unbinding rate. We can also investigate a less constrained model where binding on-rates vary between conformations, the cycle condition does not hold in any of the cycles, and both the TF and Pol can affect both the opening and closing rates by arbitrary factors (Fig. S3A).

In this case, we have not been able to achieve an analytical proof as above to show that responses are monotonic in the coherent modes, so we resorted to extensive numerical exploration to test that responses were monotonic under the constraints in Fig. S3, which correspond to coherent positive or negative regulation. When these constraints are not satisfied, non-monotonic responses can appear (Fig. S3B, "incoherent mode").

For monotonic activation (coherent positive), the constraints imply that 1) both the TF and Pol exhibit positive cooperativity (higher binding rate and lower unbinding rate when binding to or from the state where the other molecule is bound, as compared to binding and unbinding when the other is not bound in the same conformation); 2) both the TF and Pol bind more in the open conformation (on-rates higher and off-rates lower for the states in the open conformation than for any of the closed); 3) expression is higher from the states with both TF and Pol and even higher in the open conformation; 4) both TF and Pol favor the open conformation.

For monotonic repression (coherent negative), Pol binds more in the open conformation and favors it, and expresses more from the open conformation. The repressive effect of the TF comes from: 1) TF binds more in the closed conformation and it tends to favor the closed conformation; 2) TF reduces Pol binding (and vice-versa, i.e. there is negative binding cooperativity between the two); 3) when both TF and Pol are bound, the effect of the TF on the conformational transitions is stronger than that of Pol, so closing is favored.

To test that these constraints ensure monotonic responses, we sampled 1 million parameter sets for each of the two conditions. The parameter sets were sampled uniformly on a base 10 logarithmic scale, from the polytopes defined by the constraints, within the bounds (0,6). For this, we used the code in https://github.com/kmnam/convex-polytopes.git (commit 479f2e4e675803e213d44e6361f06491f336b0b7). Then, parameter values were obtained within the range of (1e-3, 1e3) after rescaling and exponentiating using base 10.

For each parameter set, monotonicity was assessed by numerically searching for zeros of the derivative of the input-output function $r^*(x)$. This was done with custom C++ code with high precision types provided by Boost/MPFR (100 digit precision was used). This code is run from Python using pybind11, and is based on the class GRFCalculations available in https://github.com/rosamc/GeneRegulatoryFunctions.git (commit 5822641c807b952ea9be3cc4e960a9ba134bcfd7a). First, $r^*(x)$ was defined as a rational function in terms of the q_i and the graph edge labels (as described in "Calculating steady states in the linear framework"). Then, for each parameter set, the numerical values of the coefficients of the rational function were obtained,

and the derivative $d(r^*(x))/dx$ calculated using the derivative formula. We searched for zeros of this derivative using the custom implementation of the Aberth-Ehrlich polynomial root-finding method available in https://github.com/kmnam/polynomials.git (commit af5a8318a6d637680033858a9b902c6d02eff613).

F. Monotonicity of the response in the transcription cycle model for TFs that only enhance or hamper the cycle. A similar procedure to that described for the non-equilibrium TF-chromatin model in SI Appendix section E.3, can be applied to show that the response to the TF in the model of Fig. 3B is monotonically increasing if $\epsilon_1 \geq 1$, $\epsilon_2 \geq 1$, $\epsilon_3 \geq 1$, $\epsilon_{1_r} \leq 1$ with at least one inequality. In this case, we work directly with the numerator of the derivative of the GRF. We collect terms with common binding, unbinding and basal rate parameters. This leaves a collection of expressions in ϵ_i, that have to be shown always positive when the above-mentioned constraints hold. We use mathematica to do so. When the inequalities are reversed, we find that monotonic repression is ensured.

G. Variations of the transcription cycle model. In order to check that the emergence of non-monotonicity in the model in Fig. 3B generalises beyond the specific details of that particular case, we explored variations of the model considering different order of the processes controlled positively and negatively by the TF, reversibility patterns, points of control of the TF, and number of states (in addition to the model with 3 states, we tested a model with 5 states). In order to refer to each model, we use a string, with general form $S\_R\_t = T\_d = d_1, d_2$ (Fig. S4A), which corresponds to the following encoding:

S is the number of states in the cycle of the model (we tested $S \in \{3, 5\}$). We consider a labelling of the states $1, 2, \cdots, S-1, S$ in a clockwise manner, such that there is an edge between i and $i+1$ (or i and 1 if $i = S$), which we refer to as transition i. As in the model in Fig. 3C, transcription rate is proportional to the flux through transition S, added for both TF bound and unbound states. To refer to a transition in the forward direction, we use subscript $_f$, and if the transition is reversible, we use subscript $_r$ to refer to the reverse transition. R is the set of reversible transitions in the cycle ($R \subseteq \{1, 2, \cdots ..., S-1\}$), and T the set of two transitions assumed to be modulated by the TF ($T = \{i_x, j_y\}$, $x, y \in \{_f, _r\}$). Finally we use "d_1, d_2" to encode the direction in which the TF affects the first transition and the second transition in T, such that $d_i = p$ if the value of that transition rate is higher when the TF is bound as compared to the basal cycle, and $d_i = m$ if the value of the transition rate is lower when the TF is bound. The model in Fig. 3B, with the parameters in panel D, corresponds to $3\_1\_t = \{2_f, 3_f\}\_d = p, m$ (Fig. S4A).

We explored the following models:

1. $3\_\{1\}\_t = \{1_f, 2_f\}\_d = m, p$
2. $3\_\{1\}\_t = \{1_r, 2_f\}\_d = p, p$
3. $3\_\{1\}\_t = \{1_r, 2_f\}\_d = m, m$
4. $3\_\{1\}\_t = \{1_r, 3_f\}\_d = p, p$
5. $3\_\{1\}\_t = \{1_r, 3_f\}\_d = m, m$
6. $3\_\{2\}\_t = \{1_f, 2_f\}\_d = p, m$
7. $3\_\{2\}\_t = \{1_f, 2_f\}\_d = m, p$
8. $3\_\{2\}\_t = \{1_f, 2_r\}\_d = p, p$
9. $3\_\{2\}\_t = \{1_f, 2_r\}\_d = m, m$
10. $3\_\{2\}\_t = \{1_f, 3_f\}\_d = m, p$
11. $3\_\{2\}\_t = \{1_f, 3_f\}\_d = p, m$
12. $3\_\{1, 2\}\_t = \{1_f, 2_f\}\_d = p, m$
13. $3\_\{1, 2\}\_t = \{1_f, 2_f\}\_d = m, p$
14. $3\_\{1, 2\}\_t = \{1_f, 3_f\}\_d = p, m$
15. $3\_\{1, 2\}\_t = \{1_f, 3_f\}\_d = m, p$
16. $5\_\{1, 2, 3, 4\}\_t = \{1_f, 2_f\}\_d = p, m$
17. $5\_\{1, 2, 3, 4\}_t = \{2_f, 4_f\}\_d = m, p$

For each model, we explored the parameter space aiming to identify particular kinds of behaviour: monotonically increasing and decreasing responses, bell-shaped responses, u-shaped responses and responses with more than one critical point. For this, we developed a score for input-output responses such that each of these kinds of responses falls into a particular region of a 2D space:

Given the steady-state response to TF $r^*(x)$, we evaluate it over the relevant range $[x_0,\ x_1]$, and consider $f(x) = log_2(r^*(x)/r^*(0))$. Then we define y_1 and y_c as follows:

$$y_1 \equiv f(x_1)$$

$$y_c \equiv \begin{cases} y_1 & \text{if } f(x) \text{ is monotonically increasing} \\ f(x_0) & \text{if } f(x) \text{ is monotonically decreasing} \\ f(x_c) & \text{if } f(x) \text{ has a single critical point at } x = x_c \\ 0 & \text{if } f(x) \text{ has more than one critical point} \end{cases}$$

Note that $f(x_0) = 0$ always. Therefore, a given response corresponds to a point in a 2D space defined by (y_c, y_1), as follows:
- on the positive y-axis: more than one critical point.
- diagonal of the positive quadrant: monotonically increasing.
- below the diagonal of the positive quadrant: bell-shaped ending at $f(x) > 0$.
- quadrant where $y_c > 0, y_1 < 0$: bell-shaped ending at $f(x) < 0$.
- on the negative y-axis: monotonically decreasing.
- quadrant where $y_c < 0, y_1 < 0$: u-shaped, ending at $f(x) < 0$.
- quadrant where $y_c < 0, y_1 > 0$: u-shaped, ending at $f(x) > 0$.

We then used a biased-sampling algorithm that we've developed in previous work (19) to explore the region of this 2D space that each model can occupy. Briefly, the 2D space is discretised into a grid, and the algorithm samples parameter sets iteratively, calculates its (y_c, y_1) value and corresponding grid cell, and iteratively modifies the parameter sets to fill more grids, until no new grid cells can be filled. We note that for $S = 5$ the exploration is very slow, so we stopped after 30 days of exploration. Parameter values for the basal cycle transitions and binding and unbinding rates were sampled within the range $[1, 10^4]$, considered in arbitrary units. For the TF-modulated transitions, the TF was assumed to modulate the basal rate a hundred fold (up or down).

We observed the emergence of non-monotonic, bell-shaped responses, in all models tested, except when the two transitions regulated by the TF start at the same cycle state (models $3\_\{1\}\_t = \{1_r, 2_f\}\_d = p, p$, $3\_\{1\}\_t = \{1_r, 2_f\}\_d = m, m$). Moreover, for model $5\_\{1, 2, 3, 4\}_t = \{2_f, 4_f\}\_d = m, p$, we also found non-monotonic u-shaped responses. Examples of non-monotonic responses for various models are shown in Fig. S4B-E, showing how very similar bell-shaped responses can be obtained by various implementations of the model.

H. Incoherent regulation through functional interference among activators. The incoherent regulatory mode of a TF, in which it acts positively on some transcriptional step(s) and negatively on other(s), could arise through various molecular mechanisms. For example, we could imagine that a TF on its own is always activating, and the negative effects arise as a result of interference with another activator. To illustrate this scenario, we expand the Pol cycle model in Fig. 3B to include a second TF, Y (Fig. S7). We assume that X and Y bind each to their own site, independently of each other, and each has a given on- and off-binding rate, the same for all the cycle states. When bound individually, TF X is assumed to enhance the first and second transitions of the cycle, from basal values $(k_{1,\emptyset},\ k_{2,\emptyset})$, to values $(k_{1,X},\ k_{2,X})$, and TF Y is assumed to enhance the second transition only. Therefore, when present alone, they always behave as activators. However, we could imagine a situation where they interfere with each other's effect on the second transition when bound together, i.e. $k_{2,\emptyset} < k_{2,\{X,Y\}} < \min(k_{2,X}, k_{2,Y})$. In this situation, Fig. S7B shows that X always behaves as an activator when bound alone (gray line) but in the presence of a sufficient concentration of Y (black line), X can behave as an activator, a repressor or cause a non-monotonic response over a fixed concentration range, with the X unbinding rate (b_X) tuning the direction of the response. We see that this is the same behaviour that we've seen in Fig. 3C with a single TF considered explicitly, when it is assumed to act in incoherent mode, suggesting that functional interference might be a plausible molecular mechanism underlying incoherent regulation.

I. Monotonicity of the average occupancy with respect to TF concentration and site number, for a TF that binds independently and with the same affinity to all sites. By the application of Eq. S4 to a regulatory system with N TF binding sites to which the TF binds independently with affinity K_T, the steady-state probability of a state with k molecules bound is given by $(K_T x)^k/Z$, where $Z = \Sigma_{i=0}^{N}\binom{N}{i}(K_T x)^i$. This yields the equation for average TF occupancy a:

$$a = \frac{\Sigma_{i=0}^{N}(i\binom{N}{i}(K_T x)^i)}{\Sigma_{i=0}^{N}(\binom{N}{i}(K_T x)^i)} \qquad [S63]$$

By the binomial theorem, the denominator becomes $(1 + K_T x)^N$. Similarly, we can simplify the numerator:

$$\sum_{i=0}^{N} i\binom{N}{i}(K_Tx)^i = \sum_{i=0}^{N} N\binom{N-1}{i-1}(K_Tx)^i = N\sum_{i=0}^{N}\binom{N-1}{i-1}(K_Tx)^i = N\sum_{j=0}^{N-1}\binom{N-1}{j}(K_Tx)^{j+1} = N(K_Tx)(1+K_Tx)^{N-1}$$

Therefore,

$$a = \frac{NK_Tx}{1+K_Tx}$$

It is easy to show that this expression is increasing both with x and N just differentiating with respect to either variable.

J. Monotonicity of the 3-state cycle model under coherent regulation when the rates are monotonous functions of the input (Fig. 3E). Let $k_1(a)$, $k_2(a)$, $k_3(a)$ and $k_{1,r}(a)$ be the transition rates of a 3-state transcription cycle model, which are functions of the equilibrium occupancy of the TF on the regulatory sequence a. The steady-state transcription rate $r(a)$ is given by:

$$r(a) = \frac{k_3(a)k_1(a)k_2(a)}{k_1(a)k_2(a) + k_1(a)k_3(a) + k_2(a)k_3(a) + k_3(a)k_{1,r}(a)} \quad \text{[S64]}$$

and the sign of $dr(a)/da$ depends on the following expression (where $'$ indicates derivative with respect to a, and the explicit dependence of a has been dropped for notational simplicity):

$$k_1^2k_2^2k_3' + k_1^2k_3^2k_2' - k_1k_2k_3^2k_{1,r}' + k_1k_3^2k_{1,r}k_2' + k_2^2k_3^2k_1' + k_2k_3^2k_{1,r}k_1' \quad \text{[S65]}$$

Therefore, under coherent positive regulation, where $k_1(a)$, $k_2(a)$ and/or $k_3(a)$ are increasing functions of a, and $k_{1,r}(a)$ is a decreasing function of a, the sign is positive and therefore only monotonic increasing responses with a are possible. As demonstrated in the previous section, a increases with site number or concentration. Therefore, $r(a)$ must be increasing with respect to site number or concentration under coherent positive regulation. For coherent negative regulation, the opposite is true, and only monotonic decreasing responses can arise.

K. Dynamic ranges of the non-monotonic responses for the model in Fig. 3B. Here we explore the dynamic ranges of the non-monotonic responses that the model in Fig. 3B can generate through random sampling of parameter sets. We consider the following parameter ranges: $a_T \in [10^{-2}, 10]$, $k_1, k_{1,r}, k_2, k_3 \in [10^{-2}, 10]$. $\epsilon_2, \epsilon_3 \in [10^{-2}, 10^2]$. $\epsilon_{1,r} = 1$ always. We considered two scenarios: $\epsilon_1 = 1$ (corresponding to equilibrium TF-Pol-DNA binding), and $\epsilon_1 \neq 1$ (corresponding to non-equilibrium binding). For each, we sampled 50000 parameter sets taking each parameter from the base-10 logarithmic range of the corresponding range (e.g. to choose a value for ϵ_2, we sample a from the uniform distribution between $[-2, 2]$ and set $\epsilon_2 = 10^a$.)

For each parameter set, we calculated the response fold change over the matrix resulting from 100 unbinding rate values logarithmically spaced between 0.01 and 10 s^{-1}, and 100 input concentration (x) points logarithmically spaced between 1 and 100 nM. For each row (unbinding rate), we assessed whether the response was non-monotonic, and if so calculated the corresponding dynamic range of the response fold change for that unbinding rate, $D_{F(x),b_T}$. We defined $D_{F(x),b_T}$ as the sum of the ratio between the maximum of the fold change over the fold change at $x = 1$, and the ratio between the maximum of the fold change over the fold change at $x = 100$.

$$D_{F(x),b_T} = D_1(b_T) + D_2(b_T) \quad \text{[S66]}$$

$$D_1(b_T) = \frac{\max_x F(x, b_T)}{F(1, b_T)} \quad \text{[S67]}$$

$$D_2(b_T) = \frac{\max_x F(x, b_T)}{F(100, b_T)} \quad \text{[S68]}$$

For each parameter set, we saved the best $D_{F(x),b_T}$. We then plotted the distribution of these values, for responses satisfying $D_1/D_2 > 1$ and $D_1/D_2 < r$, for $r = 1.5, 2, 5$. The first condition restricts the analysis to responses similar to those in Fig. 4B, where the last point is higher than the first one, whereas the second one imposes that the decrease after the peak should be less or more pronounced (less decrease after the peak with higher r). The results are shown in Fig. S8 and show that the dynamic ranges of the non-monotonic responses increase with r and for $\epsilon_1 \neq 1$, easily reaching values up to 5-7. We note that we're only randomly sampling parameter values here, and it is therefore expected that larger dynamic ranges could be obtained by finetuning parameter sets through optimisation algorithms.

Supplemental Experimental Materials and Methods

L. eGFP reporter construct for synTF experiments. The reporter construct is the same as that in (20). It consists of a single synthetic zinc finger binding site (CGGCGTAGCCGATGTCGCGC) upstream of a minimal CMV promoter (taggcgtgtacg-gtgggaggcctatataagcagagctcgtttagtgaaccgtcagatcgcctgga) driving d2EGFP (EGFP destabilized with signal peptide for fast degradation (fusion with aa 422-461 of mouse ornithine decarboxylase)).

M. synTF construct. The synTF used for transfection in qRT-PCR experiments contains the following part of the SP1 activation domain (Residues 263 – 499) [PMID: 8278363] as previously described (20): NITLLPVNSVSAATLTPSSQAVTISSSGSQES GSQPVTSGTTISSASLVSSQASSSSFFTNANSYSTTTTTSNMGIMNFTTSGSSGTNSQGQTPQRVSGLQGSDALNIQQN QTSGGSLQAGQQKEGEQNQQTQQQQILIQPQLVQGGQALQALQAAPLSGQTFTTQAISQETLQNLQLQAVPNSGPII IRTPTVGPNGQVSWQTLQLQNLQVQNPQAQTITLAPMQGVSLGQTSSSN. The SP1 activation domain is fused to an N-terminal zinc-finger binding domain with a GGGGS flexible linker and expressed under control of a ubiquitin promoter containing a 5' sv40 nuclear localization sequence, C-terminal HA and rabbit globin polyA 3' UTR.

N. PCR plasmids. Used primer sequences are (5'-3'):
b-Actin_fwd: GGCACCCAGCACAATGAAGATCAA
b-Actin_rev: TAGAAGCATTTGCGGTGGACGATG
GAPDH_fwd: ACATCGCTCAGACACCATG
GAPDH_rev: TGTAGTTGAGGTCAATGAAGGG
eGFP_fwd: AAGTTCATCTGCACCACCG
eGFP_rev: TCCCTTGAAGAAGATGGTGCG
synTFzf_fwd: TTTTCGAGAAGACA
synTFzf_rev: GCTGCTGTGGTCGG

O. TFs whose motifs were checked for their presence in the designed MPRA sequences. Cebpa, Cic, Creb1, Ddit3, Elk1, Fli1, Fos, Gata1, Gata2, Gfi1, Gfi1b, Ikzf1, Irf7, Klf1, Klf4 Lyl1, Mecom, Meis1, Meis2, Myb Myc, Nfix, Nfkb1, Nfyc, Nr2c2, Pbx1, Runx1, Rxrg, Sp1, Spdef, Spi1, Stat5a, Tcf12, Tcf3, Tfap2a, Trp53, Yy1, Zbtb7a

Supplementary Figures

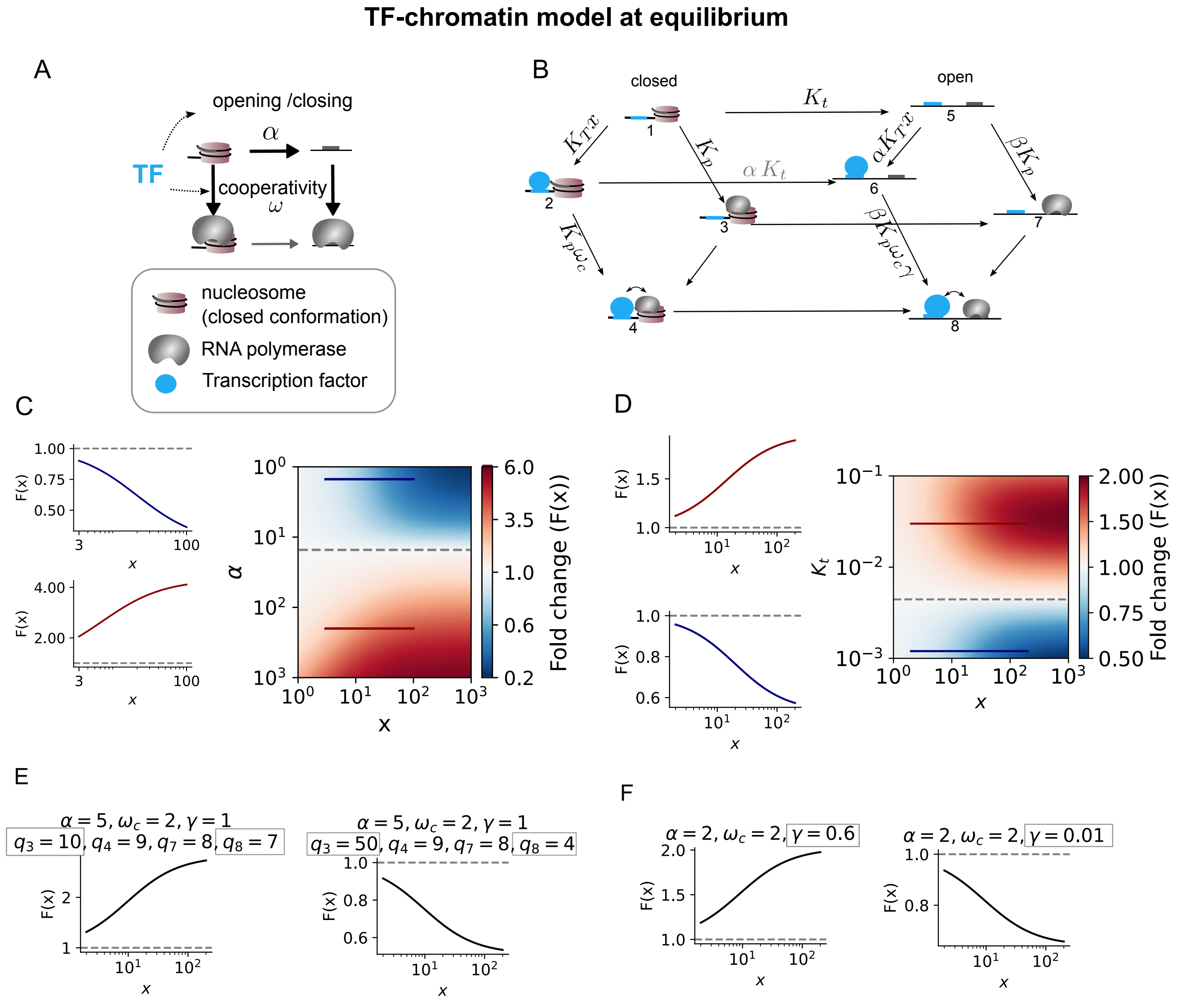

Fig. S1. The TF-chromatin model at equilibrium. A)High-level schema of the model. The promoter exists in two conformations, represented as having or not a nucleosome, and Pol has higher affinity for the open conformation than the closed. The TF can modulate Pol recruitment through effects on two processes: the conformation state, modulated by the parameter α, and binding cooperativity at a given conformation, given by the parameter ω. The text in section E discusses that the TF can modulate the transcription rates q_i, not shown in the cartoon for simplicity. B) Model at thermodynamic equilibrium. Edges are only represented in one direction (despite being reversible) with parameters the ratios between the forward and backward transition rates. At equilibrium, for the TF to have an effect on the conformation probability of the system, it must have differential binding across the two conformations, given by α. Therefore, the mechanistic effects on this process are equivalent to the affinity ratio between the two conformations. K_T is the TF association constant to the closed conformation, in units of nM^{-1}. α is the factor increase in this association constant in the open conformation. x is TF concentration, in units of nM. K_p is the Pol association constant multiplied by its concentration, and is therefore unitless. In the absence of TF and Pol, the ratio of the transition rate from the closed to the open conformation over its reverse is given by K_t. We consider $0 < K_t < 1$, so that the closed conformation is favored in the unbound state. Pol association constant to the open conformation is increased by a factor $\beta > 1$. ω_c denotes the binding cooperativity at the closed conformation, and $\omega_c \gamma$ at the open conformation. C) Monotonic activation or repression for the model in panel B in the incoherent regime ($\alpha > 1$, $\omega_c = 0.2$, $\gamma = 1$) as a function of α, the factor increase in affinity of the TF for the open conformation relative to that for the closed conformation. $K_T = 0.05, K_p = 0.01, \beta = 50, K_t = 0.01, q_3 = q_4 = q_7 = q_8 = 1$. D) Activation or repression in the incoherent regime ($\alpha = 15, \omega_c = 0.3, \gamma = 1$) as a function of K_t, which determines the basal chromatin openness. $K_T = 0.05, K_p = 0.01, \beta = 50, q_3 = 1, q_4 = 1, q_7 = 1, q_8 = 1$. E) Activation or repression with the incoherent regime given by the q_i values relative to α and ω_c. $K_T = 0.05, K_p = 0.01, \beta = 50, \alpha = 5, \omega_c = 2, \gamma = 1, K_t = 0.005$. F) Activation or repression with the incoherent regime given by inconsistent cooperativities, depending on γ. $K_T = 0.1, K_p = 0.01, \beta = 20, \alpha = 2, \omega_c = 2, K_t = 0.1, q_3 = 1, q_4 = 1, q_7 = 1, q_8 = 1$.

TF-chromatin model away from equilibrium (I)

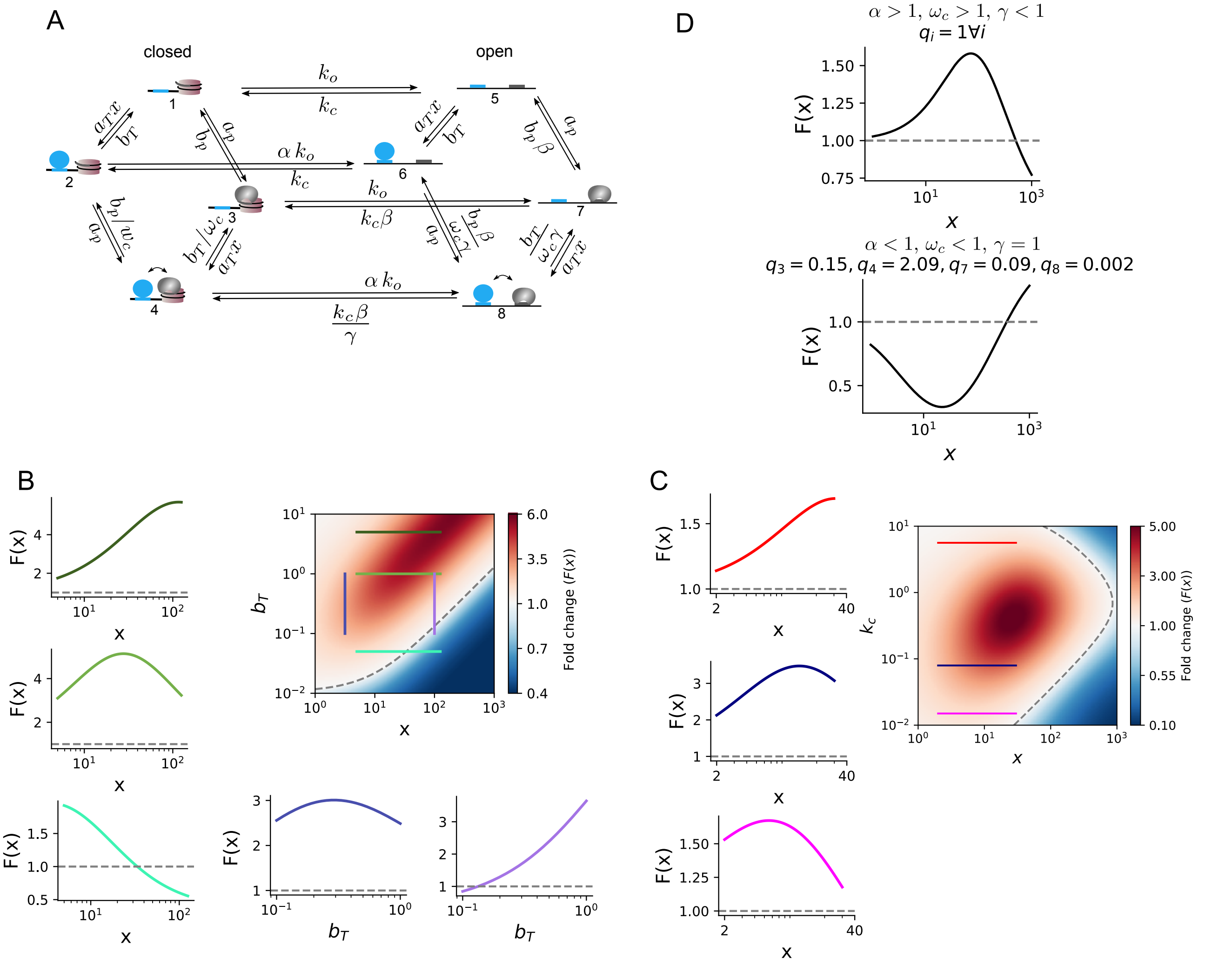

Fig. S2. The TF-chromatin model away from equilibrium. Detailed balance is broken at the cycles comprising a transition between the two conformations with the TF bound, to simulate an ATP-dependent remodelling activity recruited by the TF. B) Colormap showing the response fold change as a function of the TF unbinding rate b_T and concentration x, with lineplots corresponding to fixed concentration or unbinding rate, with the response varying as a function of the other variable. The position of the lines on the colormap is color-coded. Parameter values $a_T = 0.025$ nM^{-1} s^{-1}, $a_p = 0.01$ s^{-1}, $b_p = 100$ s^{-1}, $k_o = 0.01$ s^{-1}, $k_c = 0.25$ s^{-1}, $\beta = 0.01$, $\gamma = 1$, $\alpha = 50$, $\omega_c = 0.025$. C) Colormap of the fold change as a function of the basal closing rate k_c and corresponding lineplots showing a switch between activation and repression via a non-monotonic regime. Same parameter values as in B, with $b_T = 0.75$s^{-1}. D) Non-monotonic responses when either $\gamma \neq 1$, or $q_i \neq 1$. Parameter values (same units as in B):
Top: a_T = 0.01, b_T = 1, a_p = 0.1, b_p = 100, k_o = 0.01, k_c = 0.5, α = 5, β = 0.001, γ = 0.01, ω_c = 5, q_3 = 1, q_4 = 1, q_7 = 1, q_8 = 1.
Bottom: a_T = 0.03, b_T = 0.1, a_p = 0.022, b_p = 0.17, k_o = 0.18, k_c = 0.0033, α = 0.0077, β = 0.067, γ = 1, ω_c = 0.7.

TF-chromatin model away from equilibrium (II)

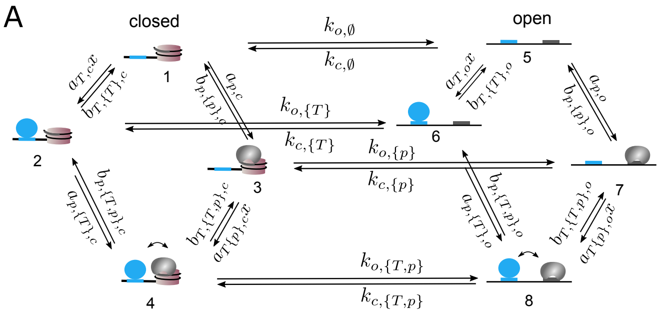

$$r(x) = q_3\,P_3(x) + q_4\,P_4(x) + q_7\,P_7(x) + q_8\,P_8(x)$$

coherent positive mode

$q_3 \leq q_4 \leq q_7 \leq q_8$

$a_{T,c} \leq a_{T,\{p\},c} \leq a_{T,o} \leq a_{T,\{p\},o}$

$b_{T,\{T\},c} \geq b_{T,\{T,p\},c} \geq b_{T,\{T\},o} \geq b_{T,\{T,p\},o}$

$a_{p,c} \leq a_{p,\{T\},c} \leq a_{p,o} \leq a_{p,\{T\},o}$

$b_{p,\{p\},c} \geq b_{p,\{T,p\},c} \geq b_{p,\{p\},o} \geq b_{p,\{T,p\},o}$

$k_{o,\{T\}} \geq k_{o,\emptyset}$

$k_{o,\{p\}} \geq k_{o,\emptyset}$

$k_{o,\{T,p\}} \geq \max\{k_{o,\{T\}}, k_{o,\{p\}}\}$

$k_{c,\{T\}} \leq k_{c,\emptyset}$

$k_{c,\{p\}} \leq k_{c,\emptyset}$

$k_{c,\{T,p\}} \leq \min\{k_{c,\{T\}}, k_{c,\{p\}}\}$

coherent negative mode

$q_7 \geq q_8 \geq q_3 \geq q_4$

$a_{T,c} \geq a_{T,\{p\},c} \geq a_{T,o} \geq a_{T,\{p\},o}$

$b_{T,\{T\},c} \leq b_{T,\{T,p\},c} \leq b_{T,\{T\},o} \leq b_{T,\{T,p\},o}$

$a_{p,o} \geq a_{p,\{T\},o} \geq a_{p,c} \geq a_{p,\{T\},c}$

$b_{p,\{p\},o} \leq b_{p,\{T,p\},o} \leq b_{p,\{p\},c} \leq b_{p,\{T,p\},c}$

$k_{o,\{T\}} \leq k_{o,\{T,p\}} \leq k_{o,\emptyset} \leq k_{o,\{p\}}$

$k_{c,\{T\}} \geq k_{c,\{T,p\}} \geq k_{c,\emptyset} \geq k_{c,\{p\}}$

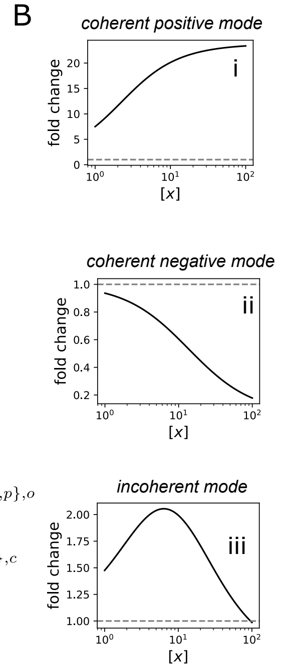

Fig. S3. Non-equilibrium TF-chromatin model with arbitrary rate relationships. A) Cartoon of the model and labelling. The conditions for coherent positive (activating) and coherent negative (repressive) modes are shown below. B) Examples for parameter sets that fulfill the conditions for coherent positive (i), coherent negative (ii) or neither (incoherent, iii). The parameter values are given in Table S1. Note the different axes ranges.

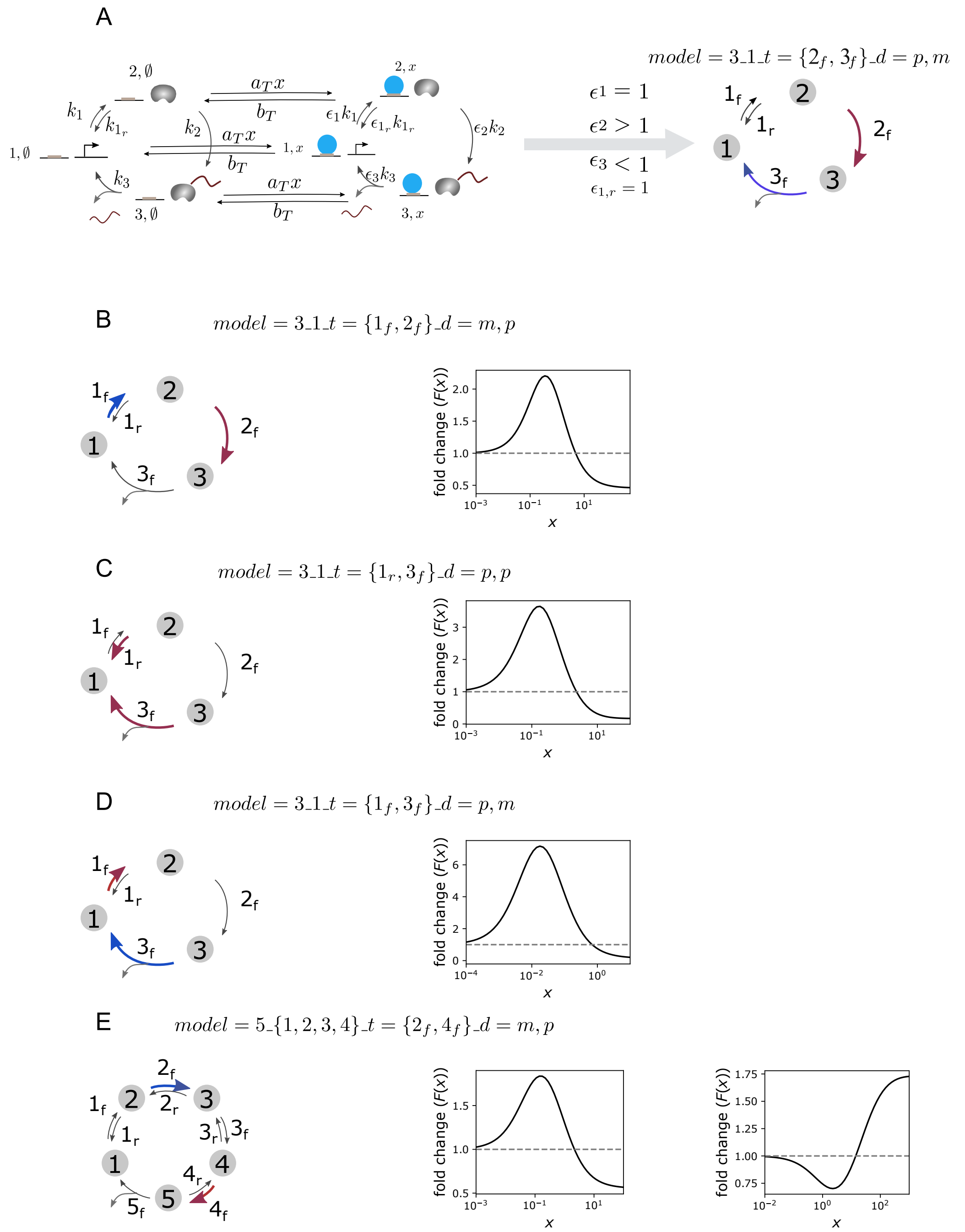

Fig. S4. Generality of the non-monotonic responses in the Pol cycle model. A) Illustration of the notation to refer to each model, as described in detail in the Supplementary text, for the model in Fig. 3B. The representation on the right is used to summarise the features of each model: number of states (3), reversible transitions (1), and transitions affected by the TF (color-coded in red for the transition accelerated by the TF, and blue for the transition slowed down). B-E) Examples of non-monotonic responses for different model implementations. Parameter values (note here the use of arbitrary units, with parameters taken from a 4-order magnitude range, from 1 to 10^4, since the goal was to test for the emergence of non-monotonicity for various points of regulation of the cycle and cycles with more states (E)): B) $k_1 = 356.52, \epsilon_1 = 0.04, k_{1_r} = 10.67, \epsilon_{1_r} = 1.00, k_2 = 36.00, \epsilon_2 = 94.62, k_3 = 338.96, \epsilon_3 = 1.00, a_T = 658.76, b_T = 201.36$. C)$k_1 = 2855.20, \epsilon_1 = 1.00, k_{1_r} = 10000.00, \epsilon_{1_r} = 85.48, k_2 = 46.46, \epsilon_2 = 1.00, k_3 = 1.00, \epsilon_3 = 17.86, a_T = 917.20, b_T = 217.86$. D)$k_1 = 1.00, \epsilon_1 = 100.00, k_{1_r} = 14.58, \epsilon_{1_r} = 1.00, k_2 = 1.00, \epsilon_2 = 1.00, k_{2_r} = 858.59, \epsilon_{2_r} = 1.00, k_3 = 34.24, \epsilon_3 = 0.01, a_T = 10000.00, b_T = 459.72$. E)left: $k_1 = 9165.58, \epsilon_1 = 1.00, k_{1_r} = 672.27, \epsilon_{1_r} = 1.00, k_2 = 1.00, \epsilon_2 = 0.21, k_{2_r} = 4.44, \epsilon_{2_r} = 1.00, k_3 = 559.60, \epsilon_3 = 1.00, k_{3_r} = 478.39, \epsilon_{3_r} = 1.00, k_4 = 3.16, \epsilon_4 = 33.64, k_{4_r} = 7.18, \epsilon_{4_r} = 1.00, k_5 = 2304.23, \epsilon_5 = 1.00, a_T = 454.67, b_T = 280.12$. right: $k_1 = 1.00, \epsilon_1 = 1.00, k_{1_r} = 103.81, \epsilon_{1_r} = 1.00, k_2 = 10000.00, \epsilon_2 = 0.12, k_{2_r} = 781.45, \epsilon_{2_r} = 1.00, k_3 = 1.00, \epsilon_3 = 1.00, k_{3_r} = 279.75, \epsilon_{3_r} = 1.00, k_4 = 10000.00, \epsilon_4 = 100.00, k_{4_r} = 482.75, \epsilon_{4_r} = 1.00, k_5 = 1.00, \epsilon_5 = 1.00, a_T = 16.31, b_T = 26.10$.

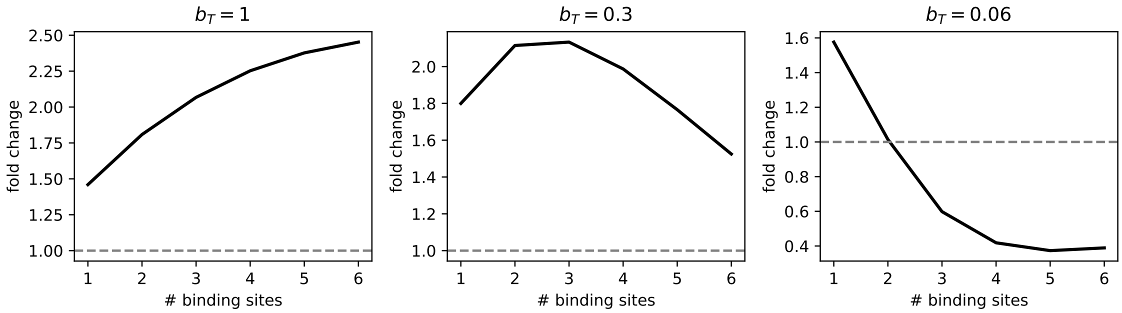

Fig. S5. Affinity-dependent activation or repression for the model in Fig. 3B extended to have one or more sites. The TF, assumed at concentration 1 nM, is assumed to bind to each site always with the same on rate (0.1 nM^{-1}s^{-1}), and unbind from each site always with the same off rate as indicated on the title of each plot (units of s^{-1}). When the TF is bound at k sites, it is assumed to increase or decrease the Pol cycle rate i by factor $\overline{\epsilon_i} = k\epsilon_i$. Parameter values: $k_1 = 0.1$ s^{-1}, $k_{1,r} = 1$ s^{-1}, $k_2 = 0.15$ s^{-1}, $k_3 = 0.15$ s^{-1}, $\epsilon_1 = 1/k$ (no effect), $\epsilon_{1,r} = 1/k$ (no effect), $\epsilon_2 = 100$, $\epsilon_3 = 0.05$.

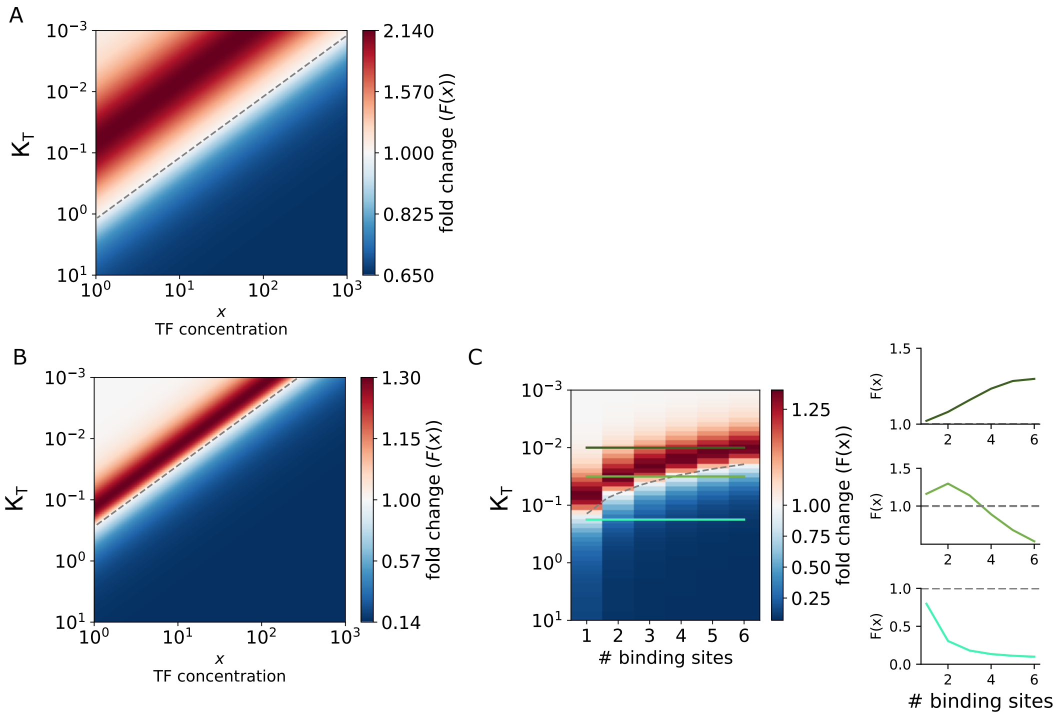

Fig. S6. Regulation of the Pol cycle model by average TF occupancy on DNA. Same parameter set as in Fig. 3F except for the differences indicated. A) Colormap showing fold change as a function of TF concentration and affinity, for a single site model. B-C) Effect of increased non-linearity in the effect of the TF on the cycle rates. Same parameter set as in A, with $h = 2$ instead of 1. B) Fold change as a function of TF concentration and affinity, for a single site model. C) Fold change as a function of affinity and number of binding sites, for $x = 2$ nM. The lineplots on the right correspond to the response at the three affinity levels marked on the colormap.

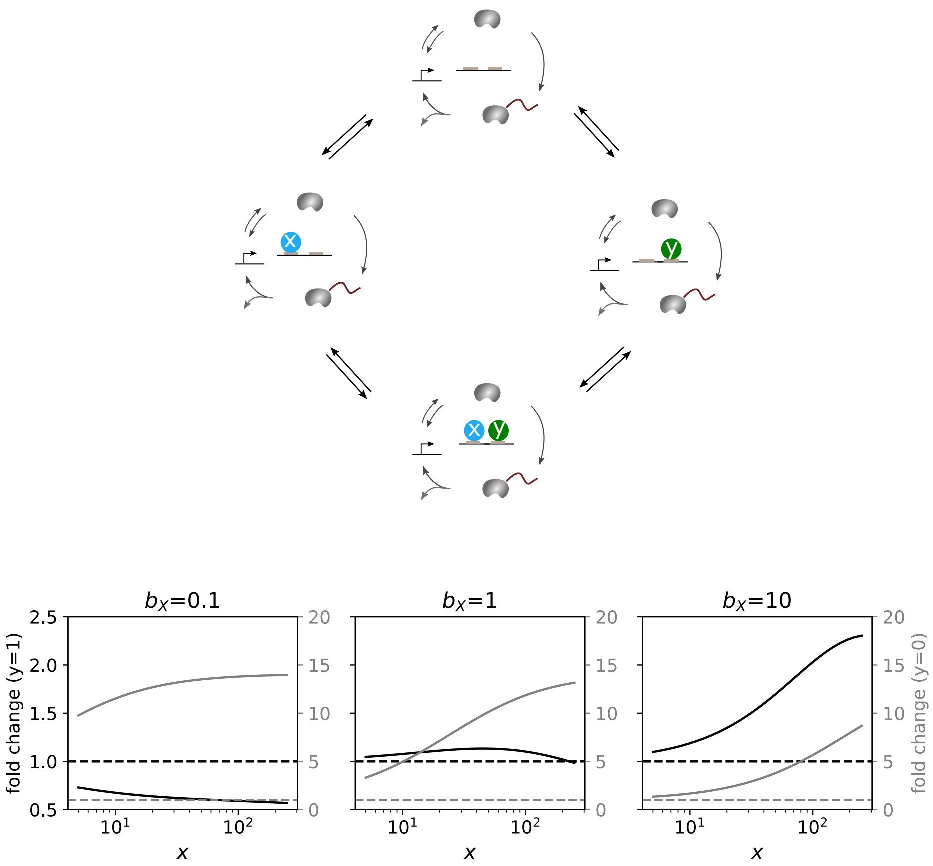

Fig. S7. Expansion of the model in Fig. 3B to account for two TFs, denoted X and Y, and duality from functional interference between them. The system has a binding and unbinding reaction connecting any given cycle state with a given binding configuration, to the same cycle state with another binding configuration, depending on whether a molecule has bound or unbound. The cartoon does not show all binding and unbinding transitions, for clarity. We assume that the binding and unbinding rate of each TF is the same regardless of which cycle state it binds to. Parameter values (same notation as in Fig. 3), with binding and unbinding rate for TF Z denoted as a_Z (nM^{-1}s^{-1}) and b_Z (s^{-1}, respectively), and $k_{i,S}$ denotes the cycle transition rate i (in units of s^{-1}) when the TFs in S are bound: $a_X = 0.04$, $a_Y = 50$, $b_Y = 1$, $k_{1,\emptyset} = 1$, $k_{1r,\emptyset} = 0.5$, $k_{2,\emptyset} = 0.25$, $k_{3,\emptyset} = 5$, $k_{1,X} = 25$, $k_{1r,X} = 0.5$, $k_{2,X} = 3.75$, $k_{3,X} = 5$, $k_{1,Y} = 1$, $k_{1r,Y} = 0.5$, $k_{2,Y} = 12.5$, $k_{3,Y} = 5.0$, $k_{1,\{X,Y\}} = 25$, $k_{1r,\{X,Y\}} = 0.5$, $k_{2,\{X,Y\}} = 0.375$, $k_{3,\{X,Y\}} = 5$.

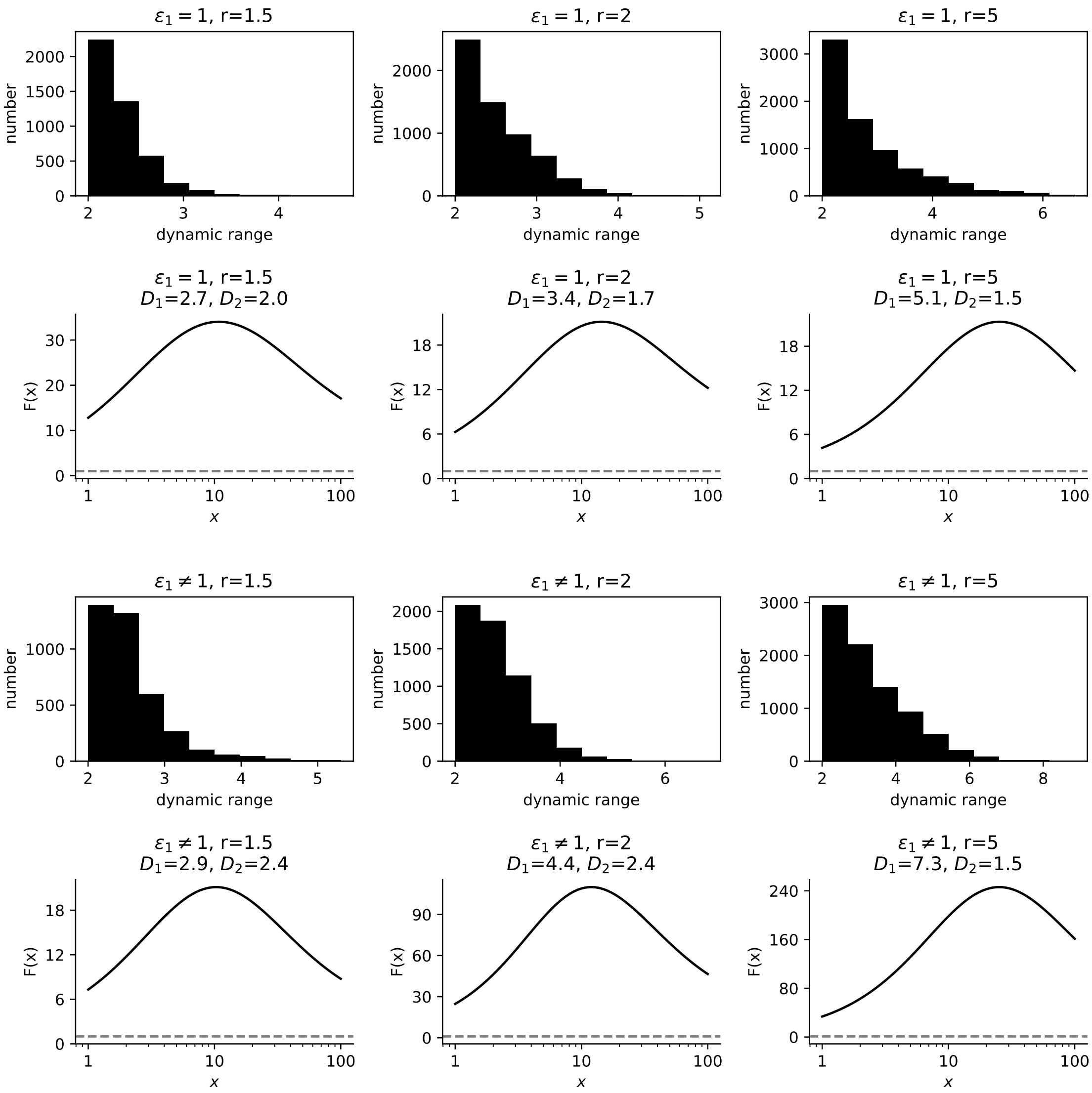

Fig. S8. Dynamic range of the non-monotonic responses for the model in Fig. 3B from randomly sampled parameter sets. The top two rows correspond to parameter sets where the TF is allowed to modulate k_2 and k_3 in the transcription cycle, whereas the bottom two rows correspond to parameter sets where the TF is allowed to also modulate k_1. r is the limit allowed for the ratio D_1/D_2, where D_1 quantifies the ratio in F(x) between the peak and first concentration point, and D_2 that between the peak and last concentration point. The dynamic range is quantified as $D_1 + D_2$, as defined in Eq. S66. The histograms show the distributions of dynamic range values for each parametric condition, and the plot below shows the curve corresponding to the highest dynamic range, with the corresponding D_1 and D_2 values. Higher r (which allows for more asymmetric responses) and $\epsilon_1 \neq 1$ allow for larger dynamic ranges.

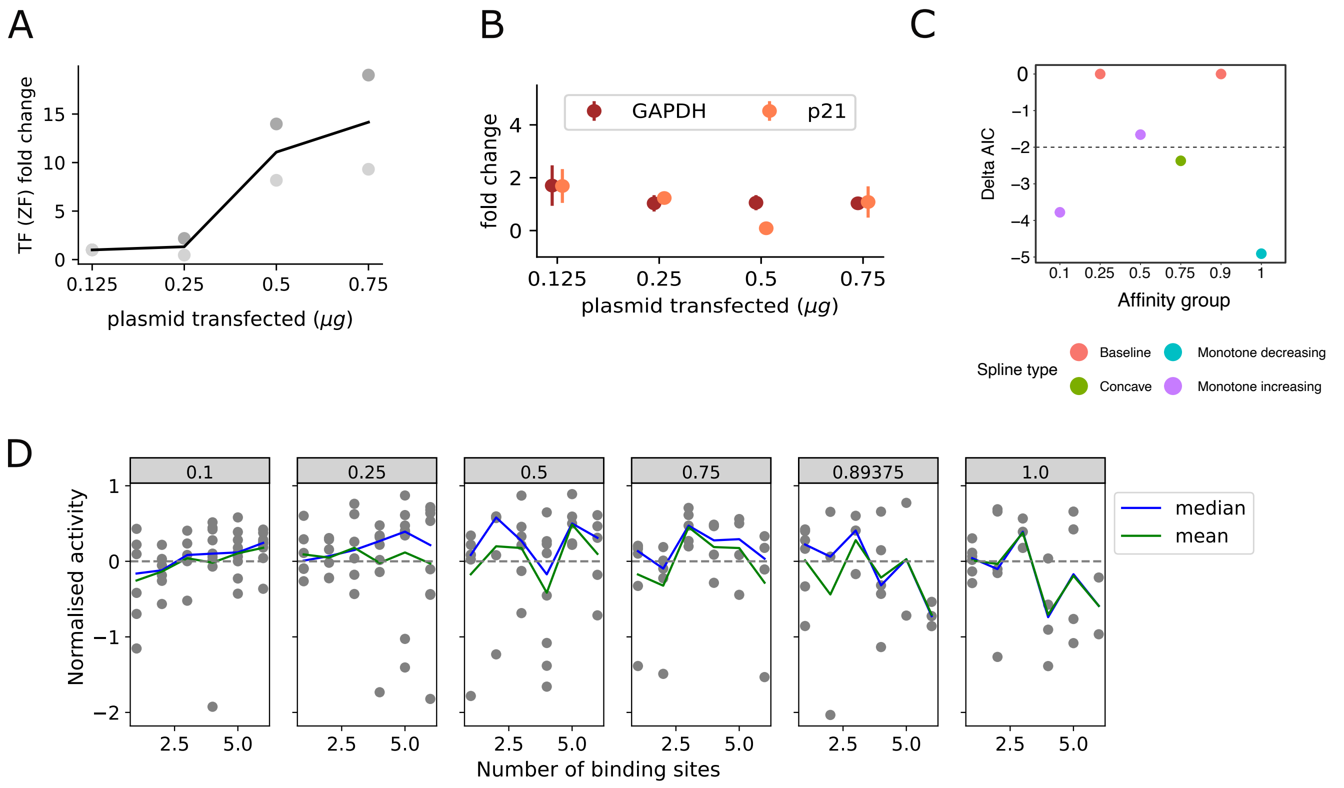

Fig. S9. Supplementary experimental panels. A) TF (ZF) mRNA fold change, relative to the lowest input condition in the plot, as measured by qPCR. Data corresponding to two biological repeats is shown as a scatter plot with two shades of gray, with the black line representing the mean. B) mRNA fold change for GAPDH and p21 over the range of synTF transfected concentrations used in these experiments, for which expression of these genes remains approximately constant, as measured by qPCR. C) Lowest AIC (Akaike Information Criterion) value difference of the fits of the data in fig. 5D with different shape constraints on P-splines, relative to a baseline horizontal fit. The dotted line set at -2 indicates the consensus for when a model is considered to be (moderately) significantly better than another (21). This analysis supports an affinity-dependent switch in the response trend, with monotonically-increasing response at the lowest affinity (0.1), concave (non-monotonic) at 0.75, and monotone decreasing at the highest affinity. D) Same data as in Fig. 5D with means and medians instead of smoothed trend. Each subplot title corresponds to the affinity group of the binding sites, with 0.1 being the lowest affinity, and 1.0 the highest.

Table S1. Parameters for the plots in Fig. S3.
Since we focus on the response fold change, the q_i parameters
are considered normalised by either the weakest or strongest of q_1, q_2, q_3, q_4.

parameter	i	ii	iii	units
$a_{T,c}$	0.50	0.08	0.30	nM^{-1}s^{-1}
$a_{T,o}$	0.94	0.02	1.35	nM^{-1}s^{-1}
$b_{T,\{T\},c}$	2.00	1.00	2.00	s^{-1}
$b_{T,\{T\},o}$	0.75	4.00	0.75	s^{-1}
$a_{p,c}$	0.01	0.01	0.01	s^{-1}
$a_{p,o}$	0.02	0.04	0.005	s^{-1}
$b_{p,\{p\},c}$	100.00	60.00	10.00	s^{-1}
$b_{p,\{p\},o}$	37.50	10.00	2.50	s^{-1}
$a_{p,\{T\},c}$	0.01	0.005	0.001	s^{-1}
$a_{p,\{T\},o}$	0.04	0.02	0.0001	s^{-1}
$b_{p,\{T,p\},c}$	75.00	180.00	10.00	s^{-1}
$b_{p,\{T,p\},o}$	18.75	20.00	12.50	s^{-1}
$a_{T,\{p\},c}$	0.625	0.04	0.9	nM^{-1}s^{-1}
$a_{T,\{p\},o}$	0.94	0.01	1.35	nM^{-1}s^{-1}
$b_{T,\{T,p\},c}$	1.50	2.00	1.50	s^{-1}
$b_{T,\{T,p\},o}$	0.38	8.00	0.38	s^{-1}
$k_{o,\emptyset}$	0.10	0.10	0.010	s^{-1}
$k_{c,\emptyset}$	1.00	1.00	10.00	s^{-1}
$k_{o,\{T\}}$	1.00	0.025	5.00	s^{-1}
$k_{c,\{T\}}$	0.10	4.00	10.00	s^{-1}
$k_{o,\{p\}}$	0.10	0.20	0.010	s^{-1}
$k_{c,\{p\}}$	1.00	0.50	10.00	s^{-1}
$k_{o,\{T,p\}}$	1.00	0.05	5.00	s^{-1}
$k_{c,\{T,p\}}$	0.10	2.00	10.00	s^{-1}
q_3	1.00	1.00	0.10	*
q_4	1.00	1.00	1.00	*
q_7	1.50	1.00	1.00	*
q_8	2.00	1.00	1.00	*

SI Dataset S1 (SuppData.csv)

Processed SP1 MPRA data.

References

1. T Gregor, DW Tank, EF Wieschaus, W Bialek, Probing the limits to positional information. *Cell* **130**, 153–164 (2007).
2. D Strebinger, et al., Endogenous fluctuations of OCT4 and SOX2 bias pluripotent cell fate decisions. *Mol. Syst. Biol.* **15**, e9002 (2019).
3. S Alamos, et al., Minimal synthetic enhancers reveal control of the probability of transcriptional engagement and its timing by a morphogen gradient. *Cell Syst.* **14**, 220–236.e3 (2023).
4. SD Mahdavi, GL Salmon, P Daghlian, HG Garcia, R Phillips, Flexibility and sensitivity in gene regulation out of equilibrium. *Proc. Natl. Acad. Sci. U. S. A.* **121**, e2411395121 (2024).
5. H Tran, et al., Precision in a rush : trade-offs between positioning and steepness of the hunchback expression pattern. *bioRxiv* (2018).
6. Dynamics database (www.mir-lab.com/dynamics-database) (2025).
7. J Gunawardena, A Linear Framework for Time-Scale Separation in Nonlinear Biochemical Systems. *PLoS ONE* **7**, e36321 (2012).
8. I Mirzaev, J Gunawardena, Laplacian Dynamics on General Graphs. *Bull. Math. Biol.* **75**, 2118–2149 (2013).
9. T Ahsendorf, F Wong, R Eils, J Gunawardena, A framework for modelling gene regulation which accommodates non-equilibrium mechanisms. *BMC Biol.* **12**, 102 (2014).
10. J Estrada, F Wong, A DePace, J Gunawardena, Information Integration and Energy Expenditure in Gene Regulation. *Cell* **166**, 234–244 (2016).
11. KM Nam, R Martinez-Corral, J Gunawardena, The linear framework: using graph theory to reveal the algebra and thermodynamics of biomolecular systems. *Interface Focus.* **12**, 20220013 (2022).
12. GK Ackers, AD Johnson, MA Shea, Quantitative model for gene regulation by lambda phage repressor. *Proc. Natl. Acad. Sci.* **79**, 1129–1133 (1982).
13. L Bintu, et al., Transcriptional regulation by the numbers: models. *Curr. opinion genetics & development* **15**, 116–24 (2005).
14. T Raveh-Sadka, M Levo, E Segal, Incorporating nucleosomes into thermodynamic models of transcription regulation. *Genome Res.* **19**, 1480–1496 (2009).
15. La Mirny, Nucleosome-mediated cooperativity between transcription factors. *Proc. Natl. Acad. Sci. United States Am.* **107**, 22534–22539 (2010).
16. R Grah, B Zoller, G Tkačik, Nonequilibrium models of optimal enhancer function. *Proc. Natl. Acad. Sci.* **117**, 31614–31622 (2020).
17. JW Biddle, R Martinez-Corral, F Wong, J Gunawardena, Allosteric conformational ensembles have unlimited capacity for integrating information. *Elife* **10**, 1–58 (2021).
18. MZ Ali, S Guharajan, V Parisutham, RC Brewster, Regulatory properties of transcription factors with diverse mechanistic function. *PLoS Comput. Biol.* **20**, e1012194 (2024).
19. R Martinez-Corral, KM Nam, AH DePace, J Gunawardena, The hill function is the universal hopfield barrier for sharpness of input–output responses. *Proc. Natl. Acad. Sci.* **121**, e2318329121 (2024).
20. R Martinez-Corral, et al., Transcriptional kinetic synergy: A complex landscape revealed by integrating modeling and synthetic biology. *Cell Syst* **14**, 324–339.e7 (2023).
21. M Greenwood, *Intermediate Statistics with R*, Open textbook library. (Montana State University), (2021).
